# Preliminary Analysis of the Formation Mechanism of Floret Color in Broccoli (*Brassica oleracea L var. italica*) Based on Transcriptomics and Targeted Metabolomics

**DOI:** 10.3390/plants14060849

**Published:** 2025-03-08

**Authors:** Qingqing Shao, Mindong Chen, Saichuan Cheng, Huangfang Lin, Biying Lin, Honghui Lin, Jianting Liu, Haisheng Zhu

**Affiliations:** 1 Fujian Key Laboratory of Vegetable Genetics and Breeding, Crops Research Institute, Fujian Academy of Agricultural Sciences, Fuzhou 350002, China; shaoqingqing807@163.com (Q.S.); 15059483846@163.com (M.C.); 15378306254@163.com (S.C.); 18060568276@163.com (H.L.); linhonghui402@163.com (H.L.); ljt338625@163.com (J.L.); 2 College of Horticulture, Fujian Agriculture and Forestry University, Fuzhou 350000, China; lby3675878@163.com

**Keywords:** broccoli, floret color, chlorophyll, anthocyanin, metabolomics, transcriptomics, candidate genes

## Abstract

Floret color is a crucial phenotypic trait in broccoli, serving as an indicator of maturity and determining its market value. However, the mechanisms underlying color variation remain unclear. In this study, six broccoli varieties with different floret colors at harvest were chosen as materials. The color difference and pigment content of florets were measured, and a combined analysis of anthocyanin-targeted metabolome and transcriptome was conducted. Our findings revealed that chlorophyll a primarily influences green, yellow-green, and light green coloration, while the wax content may contribute to gray-green coloration. The blue-green and dark blue-green coloration are regulated by both chlorophyll a and anthocyanins. Targeted metabolomics identified five anthocyanin compounds, with peonidin-3-O-glucoside as a key metabolite for blue-green coloration and delphinidin-3-O-glucoside-5-O-galactoside and peonidin-3,5-O-diglucoside for dark blue-green coloration. Transcriptomic analysis identified CHLG as a potential key regulator for yellow-green and light-green floret coloration. The blue-green coloration appears to be coregulated by a combination of genes, including the chlorophyll biosynthesis gene HEMF; anthocyanin biosynthesis genes (PAL, FLS, and UGT); and chlorophyll degradation genes (SGR, PPD, and NYC). Furthermore, upstream genes involved in both chlorophyll metabolism (CHLI, CHLD, CHLM, DVR, and CLH) and anthocyanin biosynthesis (PAL, 4CL, CHS, F3′H, and FLS) play crucial roles in determining the dark blue-green coloration of florets. Meanwhile, transcription factors of the WRKY, NAC, and TCP families are involved in chlorophyll metabolism, while those of the bHLH and MYB families participate in anthocyanin synthesis. The WGCNA identified one Hub gene for chlorophyll metabolism and two for anthocyanin synthesis. In conclusion, 35 candidate genes were identified, including 21 involved in chlorophyll metabolism and 14 in anthocyanin biosynthesis. This study provides novel insights into the molecular mechanisms of floret coloration and establishes a foundation for molecular breeding in broccoli.

## 1. Introduction

Broccoli (*Brassica oleracea var. italica*), also known as cauliflower, sprouting broccoli, or green cauliflower, is a globally consumed vegetable belonging to the *Brassica* genus in the Cruciferae family and is a variant of cabbage [1]. Its edible part is the green floret formed at the top of the main stem and lateral branches. It is highly nutritious, rich in protein, vitamin C (ascorbate), phenolic compounds, flavonoids, and other substances, and is hailed as the “crown of vegetables” [2]. Moreover, it is abundant in anticancer substances and can help prevent cardiovascular and cerebrovascular diseases, as well as leukemia, making it extremely popular among consumers [3]. With the continuous improvement of material living standards, people’s requirements for broccoli products have gradually increased. They not only demand rich nutrition but also excellent appearance quality. As the most crucial appearance quality trait of broccoli, the floret color not only has an indirect connection with the internal quality of broccoli but also directly impacts the economic value of the product [4]. The colors of broccoli florets can be roughly classified into yellow-green, light green, green, gray-green, blue-green, etc. The diversity of colors in *Brassica* plants is mainly attributed to the changes in the contents of pigments such as chlorophyll, anthocyanin, and carotenoids within the tissues, which are influenced by both intrinsic genetic factors and external environmental conditions [5].

Chlorophyll is a pigment essential for photosynthesis, mainly consisting of blue-green chlorophyll a and yellow-green chlorophyll b. At present, among *Brassica* plants, some of the key chlorophyll synthesis-related genes that play significant roles in color formation include magnesium–chelatase subunit H (CHLH), protochlorophyllide oxidoreductase (POR), chlorophyllide a oxygenase (CAO), chlorophyll synthase (CHLG), etc. [5]. For example, the mutation of CHLH causes *Brassica napus* to exhibit a yellow-green color [6]. POR might be one of the key genes responsible for reducing the chlorophyll content in the tissues of Chinese cabbage [7]. The mutation of the CAO gene leads to Chinese cabbage showing a yellow-green color [8]. Chlorophyll degradation-related genes include NON-YELLOWING 1 (NYE1), Pheophytinase (PPD), Stay-Green (SGR), etc. [5]. Wang et al. found in the stay-green mutant of Chinese cabbage that the gene *BraA03g050600.3C* encoding a magnesium chelatase was the key gene for the stay-green trait [9]. Qian et al. observed that the mutation of NYE1 in *Brassica napus* led to an increase in chlorophyll content, which was consistent with the research findings of Wang et al. on Chinese cabbage [10,11]. Currently, it has been found that transcription factors (TFs) such as TCP, NAC, and WRKY affect the color of *Brassica* plants by regulating key genes in chlorophyll metabolism [5]. Fan et al. found that *BrWRKY65* could bind to the promoters of senescence-related genes and promote the expression of chlorophyll degradation-related genes (*BrNYC1* and *BrSGR11*), resulting in the yellowing of leaves of flowering Chinese cabbage [12]. Environmental factors influencing chlorophyll metabolism primarily include temperature, light, and soil conditions. Typically, chlorophyll synthesis in plants is slower under low-temperature conditions [13]. Different light intensities, light qualities, and photoperiods have varying effects on chlorophyll metabolism in *Brassica* plants [14,15]. Additionally, elements such as nitrogen, salt, and cadmium are also environmental factors that impact chlorophyll metabolism [16,17,18].

Anthocyanin belongs to the flavonoid compounds and is a water-soluble pigment widely existing in plants. It is one of the important secondary metabolites related to plant color formation. Among *Brassica* plants, the major anthocyanin synthase genes that play crucial roles in color formation mainly include Cinnamate 4-hydroxylase (C4H), Flavanone 3-hydroxylase (F3H), Dihydroflavonol 4-reductase (DFR), and Anthocyanidin Synthase (ANS), etc. [5]. Song et al. conducted a study on four different purple cabbages and found that *BrF3H* might be the principal gene responsible for the color differences [19]. Feng et al. employed gene editing technology on white and purple kale and discovered that the *BoDFR1* gene could influence the accumulation of anthocyanin [20]. The biosynthesis of anthocyanin is regulated by transcriptional factors [21]. Currently, in *Brassica*, transcriptional factors such as R2R3-MYB, bHLH, and LBD37 have been found to have roles in regulating anthocyanin metabolism [5]. In addition, the MYB-bHLH-WD40 (MBW) complex is also an important factor in controlling the synthesis and accumulation of anthocyanin [22]. Wen et al. carried out a comparative transcriptomic analysis of green and purple broccoli and observed that the expressions of related transcriptional factors such as MYB, LBD, and ERF were significantly upregulated in purple broccoli [23]. An et al. identified MYB113 as a candidate gene that positively regulates the purple trait in mustard [24]. Yang et al. found that the R2R3-MYB transcriptional factor *BrPAP1a* was the key gene regulating the color trait in the anthocyanin-deficient mutant of a turnip [25]. Abiotic stresses, such as high temperature, low temperature, drought, salt stress, osmotic pressure, and heavy metals, can induce the accumulation of anthocyanins in plants [26,27]. Light is an essential factor for anthocyanin synthesis. Generally, strong light upregulates the expression of structural genes in the anthocyanin biosynthesis pathway, promoting anthocyanin accumulation. In contrast, under dark or low light conditions, the expression of these genes decreases, leading to reduced or no anthocyanin accumulation [28].

Carotenoids are a class of natural terpene fat-soluble pigments, most of which appear yellow, orange-red, or red [29]. In *Brassica* plants, compared to chlorophyll metabolism and anthocyanin metabolism, the number of genes associated with carotenoid metabolism that contribute to plant color variation is relatively limited. Sun et al. used CRISPR to edit *BaPDS1* and *BaPDS2*, revealing that the double mutant exhibited a pure white phenotype, while single mutants showed partial white phenotypes [30]. Fatemeh Izadpanah et al. discovered that the transcriptional level of *BoBCH* in orange cauliflower was slightly higher in both inflorescences and leaves [31]. Zhang et al. identified *BrCRTISO* as a candidate gene potentially responsible for the color difference between orange and white Chinese cabbages [32]. Solangi et al. identified *BnaA09.ZEP* as a potential candidate gene responsible for the orange coloration of petals in *Brassica napus* [33]. Carotenoid-degrading enzymes are the main enzyme families that degrade carotenoids [34]. Han et al. found that the *BoCCD4* gene was a candidate gene controlling the yellow and white petal colors of cabbage and was only expressed in white petal tissues [35]. There has been relatively little research on the transcriptional factors regulating carotenoids in *Brassica* plants. Jung et al. found that the transcriptional factors *BrA20/AN1-like*, *BrBIM1*, and *BrZFP8* were involved in the synthesis of carotenoids in white and yellow Chinese cabbages [36]. Light is the primary environmental factor influencing the carotenoid content, with different light sources having varying effects on the carotenoid levels [37]. Additionally, salt, drought, and low temperature have been identified as environmental factors that regulate carotenoid metabolism in *Brassica* plants [38].

Although previous researchers have conducted numerous studies on the floret color of broccoli, most of them mainly focused on purple broccoli and the physiological and biochemical characteristics of different floret colors [39,40]. Currently, the metabolic pathways and candidate genes responsible for floret coloring remain incompletely understood. In recent years, the combined analysis of transcriptome and metabolome has been widely applied and has become a powerful tool for identifying key genes and metabolites related to phenotypic traits in many horticultural plants [41,42]. Therefore, this study employed six broccoli varieties with distinct floret colors at the harvesting stage as experimental materials, hypothesizing that the diversity of floret colors in broccoli is governed by the differential expression of key genes in pigment metabolic pathways and the accumulation levels of their corresponding metabolites. The study aims to identify key genes and metabolites associated with floret color formation through integrated transcriptomic and targeted metabolomic analyses, elucidate the regulatory mechanisms underlying pigment metabolism in broccoli varieties with different floret colors, and provide a theoretical foundation and candidate genes for future molecular breeding strategies aimed at improving color traits in broccoli. By leveraging multi-omics data, this research will offer novel insights into the molecular mechanisms driving floret color formation and deliver valuable genetic resources for advancing breeding practices.

## 2. Results

### 2.1. Determination of Color Difference Values and Pigment Contents of Broccoli Florets

**Figure 1:**
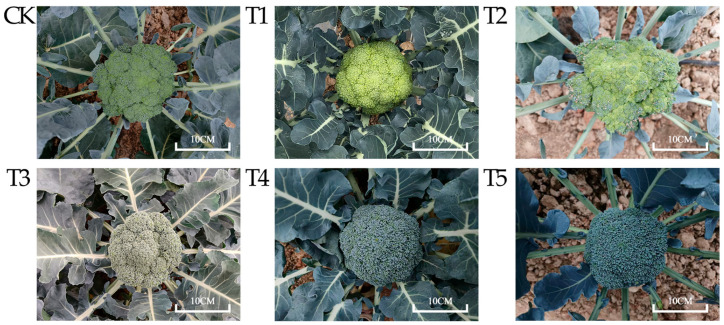
Colors of the curds of six broccoli varieties at harvest time (scale bar = 10 cm).

**Figure 2:**
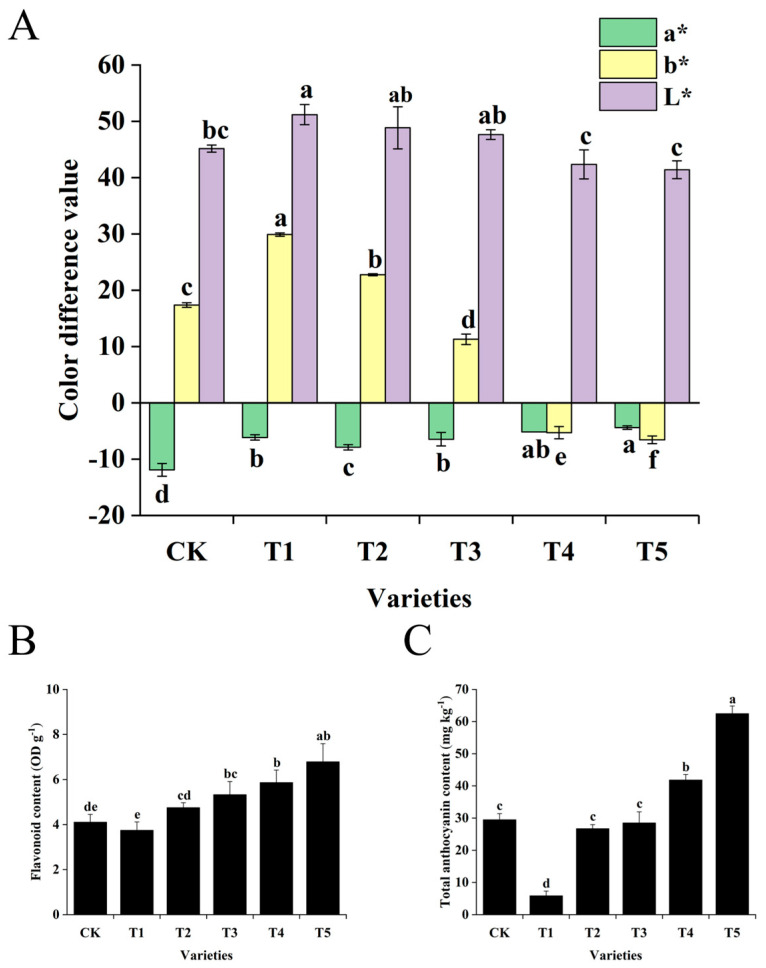
Phenotypic analysis of six broccoli varieties with different floret colors at the harvesting stage. (**A**) Color parameters of florets from six broccoli varieties. (**B**) Flavonoid content in florets of six broccoli varieties. (**C**) Anthocyanin content in florets of six broccoli varieties. Values represent the mean ± standard error (SE) of three biological replicates (n = 3). Error bars indicate the SE, and different letters above the bars denote significant differences among the six broccoli varieties at *p* < 0.05.

The floret colors of the six broccoli varieties used in this study represent characteristics typical of Chinese cultivars. Among them, CK exhibits normal green florets, T1 displays yellow-green florets, T2 features light green florets, T3 shows gray-green florets, and T4 and T5 possess blue-green florets, with the latter exhibiting a darker hue (Figure 1). The color difference values of the florets of the six broccoli varieties were measured. The results showed that the lightness values (L*) of CK (green), T1 (yellow-green), T2 (light green), T3 (gray-green), T4 (blue-green), and T5 (dark blue-green) were 45.16, 51.19, 48.85, 47.64, 42.34, and 41.40, respectively. The lightness values of the blue-green variety T4 and the dark blue-green variety T5 were the lowest, indicating that the floret colors of T4 and T5 were darker than those of the other varieties. The range of the red-green values (a*) of the floret of the six broccoli varieties was −11.91 to −4.41, and the greenness of CK with a green floret was the highest. The range of the yellow-blue values (b*) was −6.58 to 29.89. The yellowness of T1 with a yellow-green floret was the highest, and the blueness of T5 with a dark blue-green floret was the highest (Figure 2A).

The chlorophyll content and carotenoid content of the florets of the six broccoli varieties were determined. The results indicated that the total chlorophyll content in the broccoli florets increased with the deepening of the green color. T1 had the lowest content at 0.07 mg g^−1^ FW, and T5 had the highest content at 0.62 mg g^−1^ FW. The total chlorophyll content of CK was significantly higher than that of T1, higher than that of T2 and T3 without significant differences, and significantly lower than that of T5 and T4. The total chlorophyll content of T5 was significantly higher than that of T4. The contents of chlorophyll a, chlorophyll b, and carotenoids were consistent with the change trend of the color depth of the broccoli florets. The ratio of carotenoids to chlorophyll in the broccoli florets ranged from 0.11 to 0.16, suggesting that the chlorophyll content in the florets was significantly higher than that of carotenoids and was the main pigment component. Moreover, the ratio of chlorophyll a to chlorophyll b ranged from 2.10 to 2.54, indicating that chlorophyll a was the main component of chlorophyll in the florets (Table 1).

The flavonoid and anthocyanin contents of the florets of the six broccoli varieties were determined. The results showed that both the flavonoid content and the anthocyanin content of T5 were the highest, reaching 6.79 OD g^−1^ and 62.46 mg kg^−1^, respectively, while T1 had the lowest contents, which were 0.10 OD g^−1^ and 5.83 mg kg^−1^, respectively. The flavonoid content of CK was significantly lower than that of T5, T4, and T3 and had no significant differences from T1 and T2. The anthocyanin content of CK was significantly higher than that of T1, significantly lower than that of T5 and T4, and had no significant differences from T2 and T3. The flavonoid content and the anthocyanin content of T5 were both significantly higher than those of T4 (Figure 2B,C).

The above results suggest that the color difference values and pigment contents of the broccoli florets were basically consistent with the color changes of the florets. The floret colors of CK (green), T1 (yellow-green), and T2 (light green) might be mainly related to chlorophyll a and independent of the carotenoid content, while the floret colors of T5 (dark blue-green) and T4 (blue-green) might be mainly determined by the combined effects of the chlorophyll a content and the anthocyanin content.

### 2.2. Metabolic Differences Among Florets of Different Broccoli Varieties

**Figure 3:**
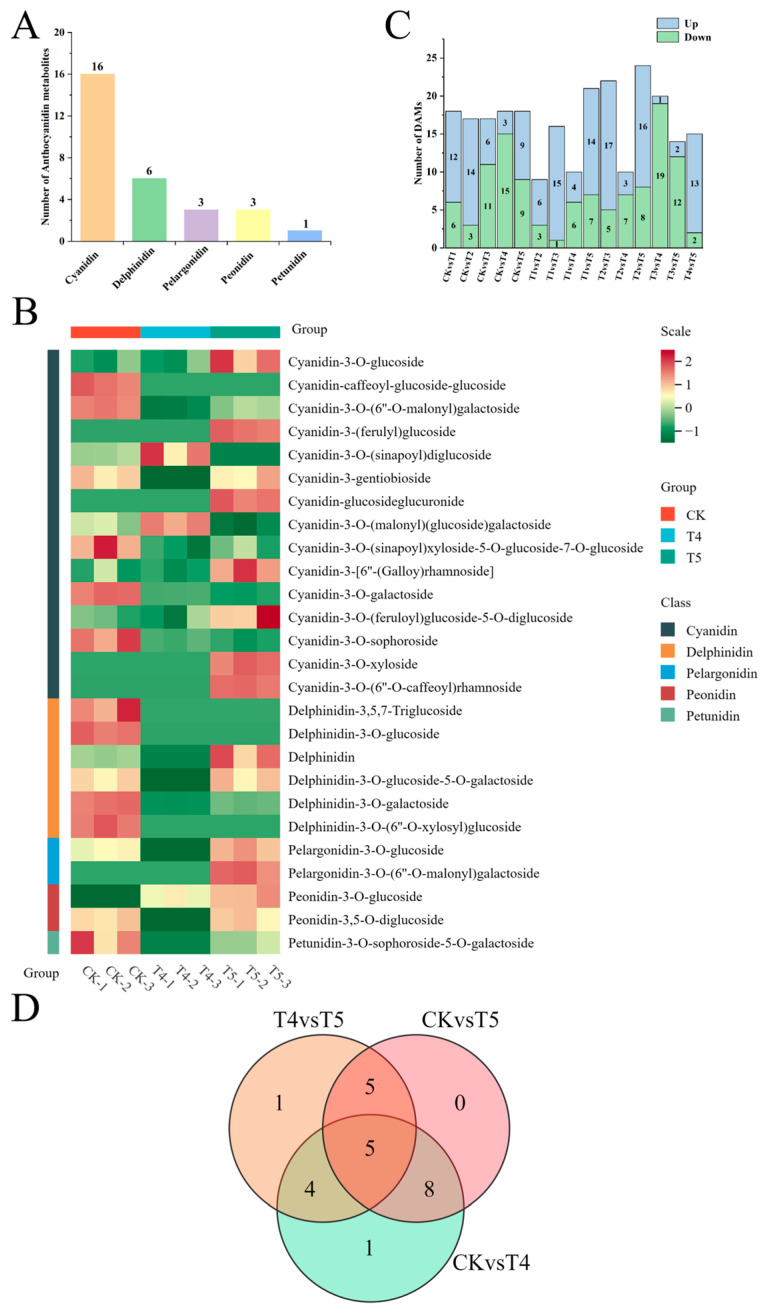
Metabolomic analysis of six broccoli varieties with different floret colors. (**A**) Classification and statistical analysis of all detected metabolites. (**B**) Heat map analysis of the relative contents of all metabolites in CK, T4, and T5. (**C**) A stacked bar plot shows the number of differentially accumulated metabolites, including upregulated and downregulated metabolites, across six broccoli varieties. (**D**) A Venn diagram illustrates overlapping and variety-specific differential metabolites among comparison groups, including CK vs. T4, CK vs. T5, and T4 vs. T5.

To further analyze the differences in anthocyanin metabolites among different varieties, liquid chromatography-tandem mass spectrometry (LC-MS/MS) technology was employed to detect anthocyanin compounds in the florets of broccoli with different colors. A total of five types of anthocyanin compounds were identified from these six groups of samples, including 16 kinds of cyanidin, 6 kinds of delphinidin, 3 kinds of pelargonidin, 3 kinds of peonidin, and 1 kind of petunidin (Figure 3A). Peonidin-3-O-glucoside was not detected in the green (CK), yellow-green (T1), and light green (T2) florets. Cyanidin-3-O-(6″-O-malonyl) galactoside was not detected in the dark green (T3) florets. Based on the concentrations of the anthocyanin metabolites, a cluster analysis of the samples was carried out. The relative contents of the identified anthocyanins varied significantly among the three broccoli varieties (Figure 3B). In the blue-green (T4) and dark blue-green (T5) florets, the contents of most cyanidin, delphinidin-3-O-glucoside-5-O-galactoside, pelargonidin, and peonidin-3,5-O-diglucoside in T5 were significantly higher than those in T4. Notably, pelargonidin-3-O-(6″-O-malonyl) galactoside was only present in T5. In general, most of the identified anthocyanins had a higher content in T5 (Figure 3B).

Using VIP ≥ 1, fold change ≥ 2, or fold change ≤ 0.5 as the thresholds, differentially accumulated metabolites (DAMs) were further identified in the comparison combinations. A total of 18 DAMs were identified in both the CK vs. T4 and CK vs. T5 combinations, but the number of upregulated metabolites in the CK vs. T5 combination was greater than that in the CK vs. T4 combination; a total of five DAMs were identified in the CK vs. T4 vs. T5 combination (Figure 3D). These anthocyanins might be the metabolites that affect the florets, making them blue-green. A total of 15 DAMs were identified in the T4 vs. T5 combination. These anthocyanins might be the metabolites that affect the florets, making them dark blue-green (Figure 3C).

### 2.3. Overview of the Transcriptome Data of Six Broccoli Varieties

Transcriptome sequencing was carried out on the florets of six broccoli varieties, and a total of 18 cDNA libraries were constructed, with three biological replicates for each variety. The average number of raw reads per sample was 70,930,352. After removing adapters and low-quality data from the original sequencing data, the average number of clean reads per sample was 66,396,493. Further statistics on high-quality bases and the GC content revealed that the Q20, Q30, and GC contents of the clean reads were 98.19–98.85%, 95.00–95.95%, and 46.79–47.61%, respectively. When the clean reads were aligned to the broccoli reference genome, the alignment rate was 91.02–93.47%. The above results indicate that the transcriptome data were of high quality and could be used for subsequent experimental analysis (Supplementary Table S1).

To determine the differences between each group of samples and the magnitude of variations within the groups, principal component analysis (PCA) was performed among the 18 samples. The distances between the CK, T1, and T2 samples and between the T4 and T5 samples were relatively close, suggesting small differences between these sample groups. The distances between the CK, T1, and T2 samples group, the T4 and T5 samples group, and the T3 samples were relatively far, indicating large differences between these sample groups. In addition, the three samples within the same group were clustered together, demonstrating good sample repeatability (Supplementary Figure S1).

### 2.4. Screening of Differentially Expressed Genes in Broccoli Florets

**Figure 4:**
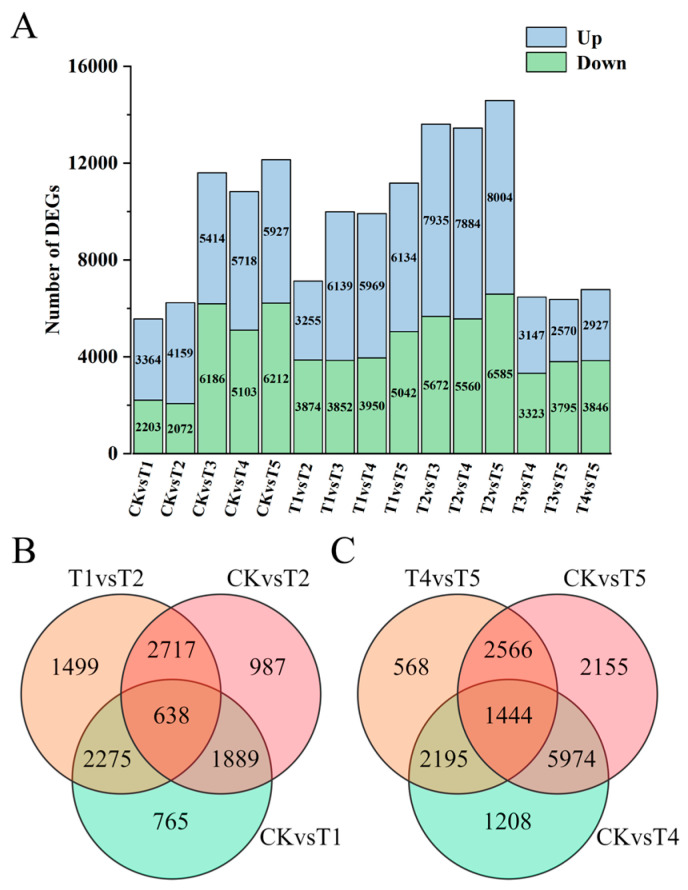
Analysis of differentially expressed genes among six broccoli varieties. (**A**) A stacked bar plot shows the number of DEGs, including upregulated and downregulated genes, across six broccoli varieties; (**B**) a Venn diagram illustrates overlapping and variety-specific DEGs among comparison groups, including CK vs. T1, CK vs. T2, and T1 vs. T2; and (**C**) a Venn diagram displays overlapping and variety-specific DEGs among comparison groups, including CK vs. T4, CK vs. T5, and T4 vs. T5.

In total, 24,230 differentially expressed genes (DEGs) were identified across 15 comparison combinations. Specifically, there were 5567 DEGs in the CK vs. T1 combination and 6231 DEGs in the CK vs. T2 combination. Notably, the number of upregulated genes in the CK vs. T2 combination surpassed that in the CK vs. T1 combination (Figure 4A). A set of 638 genes exhibited differential expression in the CK vs. T1 vs. T2 combination, implying that these DEGs might potentially harbor genes responsible for regulating the florets to display green, yellow-green, and light green colors (Figure 4B). The 11,600 DEGs in the CK vs. T3 combination could potentially include genes that govern the florets to exhibit green and gray-green hues. In the T4 vs. T5 combination, 6773 DEGs were detected, which might contain genes that modulate the florets to take on a dark blue-green color. Moreover, the CK vs. T4 combination and the CK vs. T5 combination had 10,821 and 12,139 DEGs, respectively, with the number of upregulated genes in the CK vs. T5 combination being greater than that in the CK vs. T4 combination (Figure 4A). Additionally, 1444 genes showed differential expression in the CK vs. T4 vs. T5 combination, suggesting that these DEGs might incorporate genes that regulate the florets to be green and blue-green (Figure 4C).

### 2.5. Functional Annotation of Differentially Expressed Genes in Broccoli Florets

**Figure 5:**
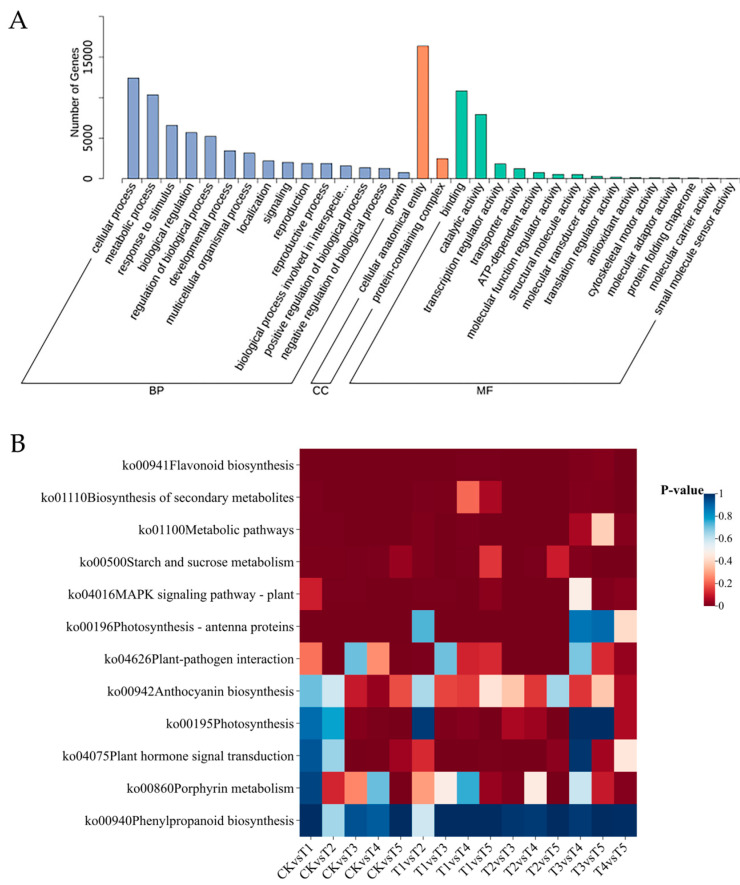
Functional annotation of differentially expressed genes in broccoli curds. (**A**) GO classification map of all DEGs. (**B**) KEGG enrichment heat map of all DEGs.

All the DEGs were mapped to the GO database. The GO classification results revealed that the DEGs were widely distributed across three functional groups: biological process, molecular function, and cellular component (Figure 5A). In the biological process, the cellular process functional group had the highest enrichment of DEGs, followed by the metabolic process and response to stimulus. Among the cellular components, cell anatomical entity and protein-containing complex were the main subgroups with enriched DEGs, while, in the molecular function, the DEGs were mainly enriched in binding and catalytic activity (Figure 5A). The GO classification of the DEGs in the CK vs. T1 vs. T2, CK vs. T3, CK vs. T4 vs. T5, and T4 vs. T5 combinations showed similar results (Supplementary Figure S2). The DEGs in both the CK vs. T1 vs. T2 and CK vs. T3 combinations were enriched in the phenylpropanoid metabolic process and phenylpropanoid biosynthesis entries. In addition, the DEGs in the CK vs. T3 combination were also enriched in entries such as photosynthesis light-harvesting, photosynthesis light-harvesting in photosystem I, and the reaction center of photosystem I. Notably, most of the DEGs were enriched in the entry of the cell response to fatty acids. However, the other combinations did not show such enrichment (Supplementary Figure S3).

Regarding the KEGG annotation results, 145 KEGG metabolic pathways were enriched for all the DEGs in the 15 comparison combinations. Most of the DEGs were mainly distributed in the flavonoid biosynthesis pathway, metabolic pathways, and biosynthesis pathway of secondary metabolites. They were also enriched in pathways such as photosynthetic antenna proteins, anthocyanin synthesis, photosynthesis, porphyrin and chlorophyll metabolism, and phenylpropanoid biosynthesis (Figure 5B). The KEGG enrichment analysis results of the DEGs in the CK vs. T1 vs. T2, CK vs. T3, CK vs. T4 vs. T5, and T4 vs. T5 combinations indicated that the DEGs in these four comparison combinations were commonly and significantly enriched in the metabolic pathway, biosynthesis pathway of secondary metabolites, and flavonoid metabolism pathway (Supplementary Figure S4). In the CK vs. T3 combination, there was also significant enrichment in pathways such as photosynthetic antenna proteins, linolenic acid metabolism, fatty acid biosynthesis pathway, and biosynthesis of flavones and flavonols. In the CK vs. T4 vs. T5 and T4 vs. T5 combinations, it was found that DEGs were significantly enriched in the porphyrin and chlorophyll biosynthesis pathway (Supplementary Figure S4).

### 2.6. Identification of Differentially Expressed Genes Related to Chlorophyll Metabolism

**Figure 6:**
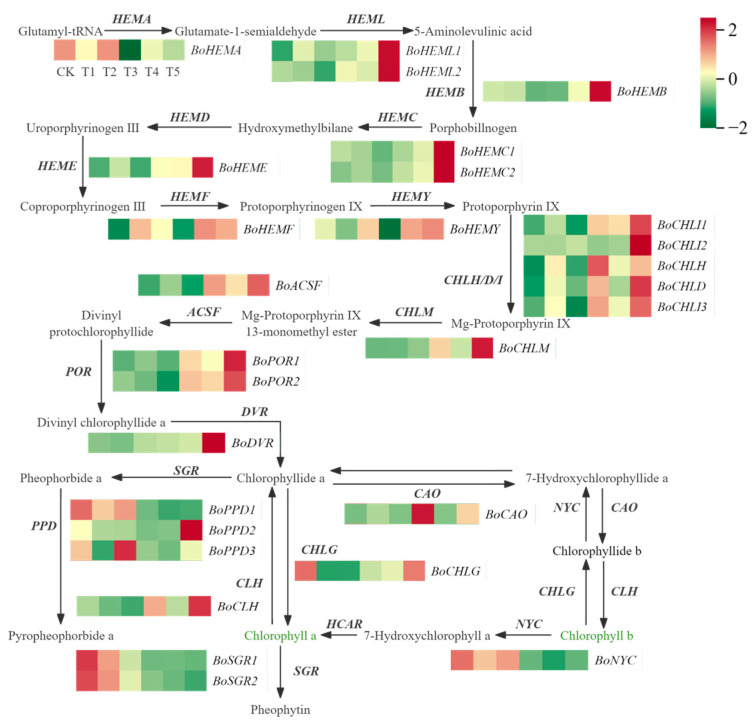
Differential expression of genes related to the chlorophyll metabolic pathway in broccoli.

To explore the role of genes related to chlorophyll metabolism in the formation of color diversity in broccoli florets, the DEGs associated with the porphyrin and chlorophyll metabolism pathways were investigated. A total of 28 DEGs related to the chlorophyll metabolism pathway were identified (Figure 6) (Supplementary Table S2). Compared to CK, the expression levels of the *BoCHLG* gene in T1 and T2 were significantly downregulated; in T4 and T5, the expression level of the *BoHEMF* gene was significantly upregulated, while the expression levels of the *BoSGR1*, *BoSGR2*, *BoPPD1*, and *BoNYC* genes were significantly downregulated; compared to T5, the expression levels of the *BoCHLI2*, *BoCHLD*, *BoCHLM*, *BoDVR* and *BoCLH* genes in T4 were significantly downregulated (Figure 6).

In conclusion, the *BoCHLG* chlorophyll synthesis gene might affect the accumulation of chlorophyll in CK, T1, and T2; the *BoHEMF* chlorophyll synthesis gene and the *BoSGR1*, *BoSGR2*, *BoPPD1*, and *BoNYC* chlorophyll degradation genes might affect the accumulation of chlorophyll in CK, T4, and T5; the *BoCHLI2*, *BoCHLD*, *BoCHLM*, *BoDVR*, and *BoCLH* chlorophyll synthesis genes might affect the accumulation of chlorophyll in T5 and T4.

### 2.7. Identification of Differentially Expressed Genes in Anthocyanin Biosynthesis

**Figure 7:**
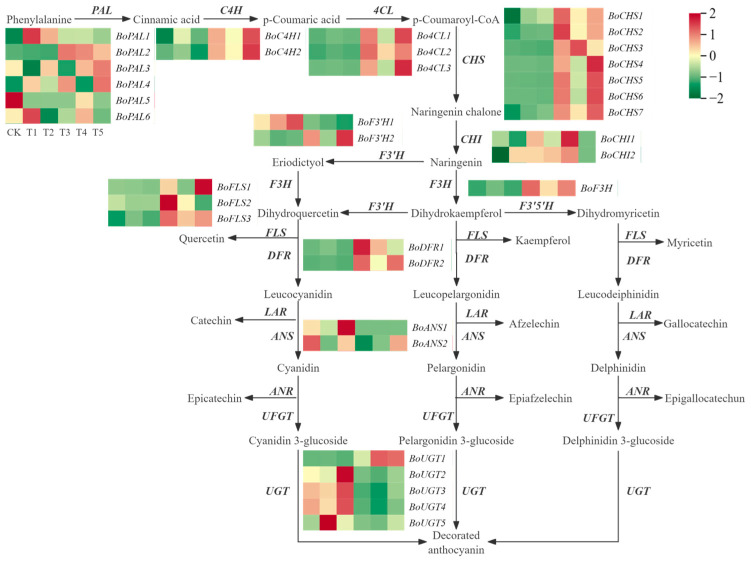
Differential expression of genes related to the anthocyanin metabolic pathway in broccoli.

To investigate the relationship between the formation of broccoli floret color and the expression of genes related to anthocyanin synthesis, the DEGs associated with the phenylpropanoid biosynthesis pathway, flavonoid biosynthesis pathway, and anthocyanin biosynthesis pathway were studied. A total of 35 DEGs related to the anthocyanin metabolism pathway were identified (Figure 7) (Supplementary Table S3). Compared to CK, the expression levels of the *BoPAL2, BoPAL4*, C4H, 4CL, CHS, *BoF3H*, *BoFLS3*, and DFR genes in T3 were significantly upregulated, while the expression level of the *BoANS2* gene was significantly downregulated; in T4 and T5, the expression levels of the *BoPAL2*, *BoFLS3* and *BoUGT1* genes were significantly upregulated; compared to T4, the expression levels of the *BoPAL4*, *Bo4CL3*, *BoCHS4*, *BoF3′H2*, and *BoFLS1* genes in T5 were significantly upregulated (Figure 7).

In summary, the *BoPAL2*, *BoPAL4*, C4H, 4CL, CHS, *BoF3H*, *BoFLS3*, and DFR genes may influence anthocyanin accumulation in CK and T3. The *BoPAL2*, *BoFLS3*, and *BoUGT1* genes may affect anthocyanin accumulation in CK, T4, and T5. Additionally, the *BoPAL4*, *Bo4CL3*, *BoCHS4*, *BoF3′H2*, and *BoFLS1* genes may contribute to the differences in anthocyanin accumulation between T5 and T4.

### 2.8. Identification of Transcription Factors Related to Chlorophyll Metabolism and Anthocyanin Biosynthesis

**Figure 8:**
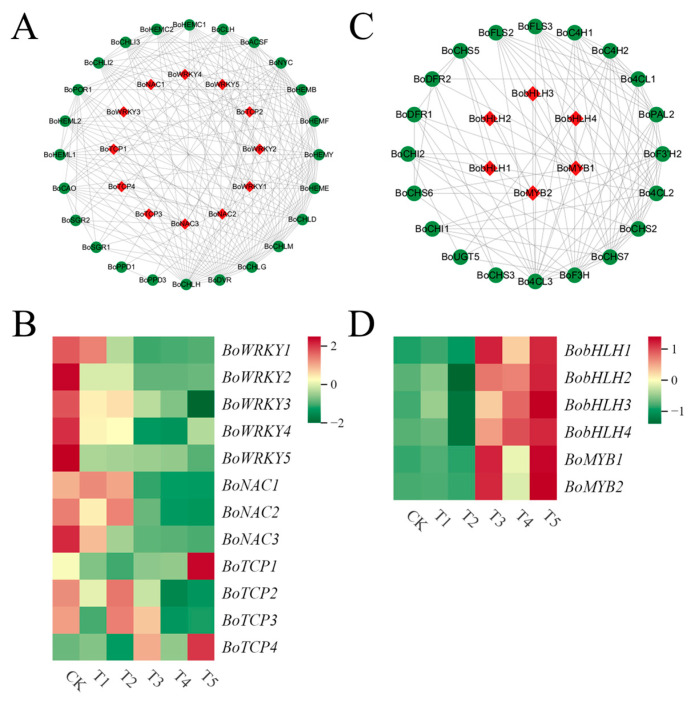
Analysis of the transcription factors related to chlorophyll metabolism and anthocyanin synthesis in broccoli curds. (**A**) Protein–protein interaction network analysis diagram of transcription factors and DEGs related to chlorophyll metabolism. (**B**) Heat map of the expression levels of DEGs in the WRKY, TCP, and NAC families. (**C**) Protein–protein interaction network analysis diagram of transcription factors and DEGs related to anthocyanin synthesis. (**D**) Heat map of the expression levels of DEGs in the MYB and BHLH families.

To explore the transcription factors involved in chlorophyll metabolism and anthocyanin biosynthesis in broccoli florets, this study analyzed the transcription factors of all DEGs through the transcriptome data of broccoli florets. Eventually, a total of 3903 transcription factors in broccoli florets were identified. Among all these transcription factors, by searching the relevant literature, the transcription factor families related to chlorophyll metabolism and anthocyanin synthesis were screened. The transcription factors of the NAC, WRKY, and TCP families, as well as the MYB and bHLH families, were regarded as putative transcription factors associated with chlorophyll metabolism and anthocyanin biosynthesis. To further investigate the relationships between these putative transcription factors and the structural enzymes in the chlorophyll metabolism and anthocyanin synthesis pathways, protein–protein interaction network analyses were carried out on the protein sequences of these putative transcription factors and the DEGs related to chlorophyll metabolism and anthocyanin synthesis (Figure 8A,C) (Supplementary Table S4).

In the protein–protein interaction network, a total of 12 transcription factors, interacted with the chlorophyll synthesis structural enzymes (Figure 8A and Table 2). The expression levels of *BoWRKY2* and *BoWRKY5* in CK were significantly higher than those in T1 and T2 and also significantly higher than those in T4 and T5. The expression levels of NAC homologous DEGs (three in total) and *BoTCP2* and *BoTCP3* in CK were significantly higher than those in T4 and T5. The expression levels of *BoTCP1* and *BoTCP4* in T5 were significantly higher than those in T4 (Figure 8B). A total of 6 transcription factors interacted with the anthocyanin synthesis structural enzymes (Figure 8C and Table 2). The expression levels of *BoMYB2* and *BobHLH1* in T3 were significantly higher than those in CK, and the expression levels of *BoMYB1* and *BoMYB2* in T5 were significantly higher than those in T4 (Figure 8D). The expression levels of *BobHLH2* and *BobHLH3* in T4 and T5 were significantly higher than those in CK.

### 2.9. Construction of the Regulatory Network for Chlorophyll and Anthocyanin Synthesis in Broccoli Florets

**Figure 9:**
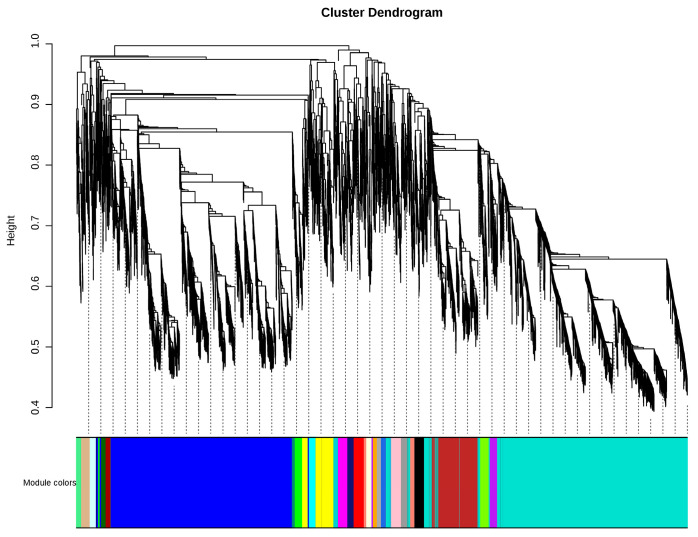
In the weighted gene co-expression network analysis, the clustering dendrogram reveals 26 gene co-expression modules.

**Figure 10:**
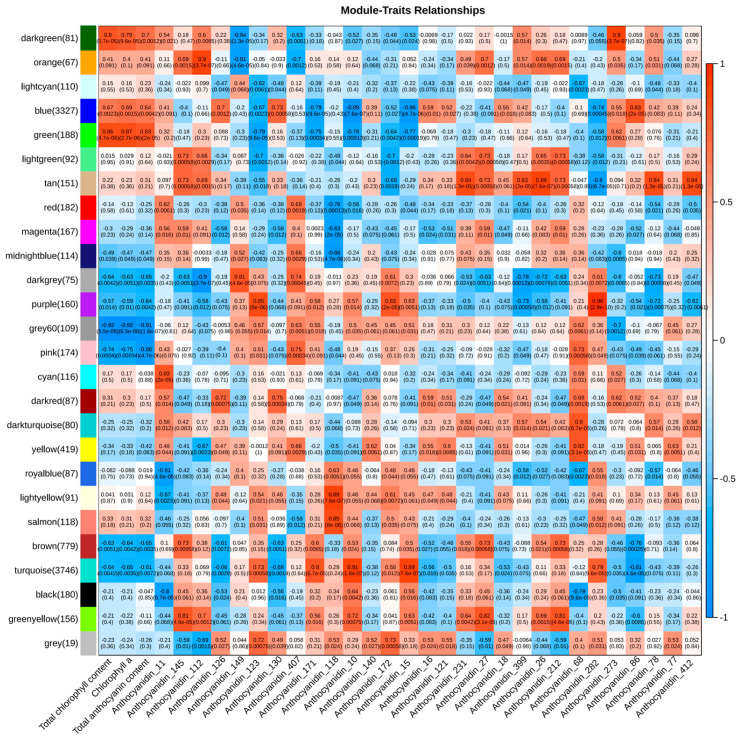
Heat map of the correlations between modules and total chlorophyll content, chlorophyll a content, total anthocyanin content, and five anthocyanin compounds.

To investigate the regulatory network of chlorophyll and anthocyanin synthesis in broccoli florets, the WGCNA was carried out using all DEGs. A total of 10,876 non-redundant DEGs were clustered into 26 modules marked with different colors (Figure 9). Through correlation analysis between the co-expression modules and the total chlorophyll content, chlorophyll a content, total anthocyanin content, and five types of anthocyanin compounds, it was found that the green module had a positive correlation with anthocyanin content, and both the dark green and green modules had positive correlations with the chlorophyll content and chlorophyll a content (Figure 10). The results of the KEGG enrichment analysis showed that the genes in the dark green module were enriched in metabolic pathways, lysosomes, and porphyrin and chlorophyll metabolism pathways, while the genes in the green module were enriched in lysosomes, metabolic pathways, and photosynthesis pathways (Supplementary Figure S5). To explore the expression patterns of the genes in these modules, a heat map analysis was performed using the FPKM values of the genes in these modules. The heat map results of the dark green module demonstrated that the co-expressed genes only had a relatively high expression level in T5, showing significant sample specificity. The heat map results of the green module indicated that the co-expressed genes had relatively high expression levels in T4 and T5 (Supplementary Figure S5).

**Figure 11:**
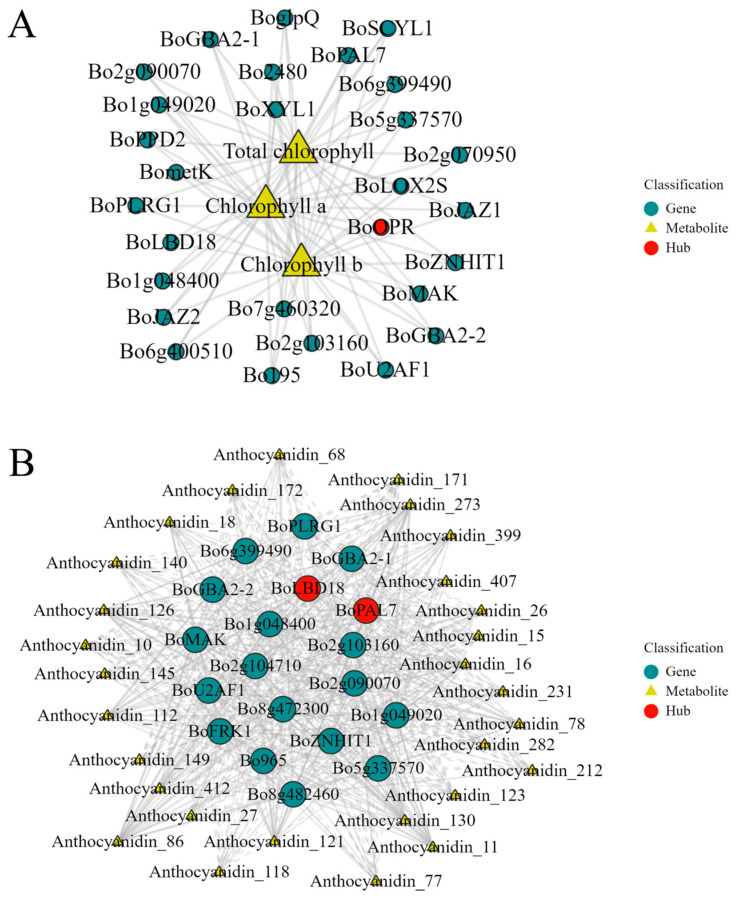
Co-expression network analysis between Hub genes and metabolites in chlorophyll metabolism and anthocyanin synthesis. (**A**) Interaction network diagram between Hub genes in the gray-green and green modules and the contents of total chlorophyll, chlorophyll a, and chlorophyll b. (**B**) Interaction network diagram between Hub genes in the green module and five anthocyanin compounds.

To further define the relationships among genes within the modules and identify Hub genes, a GS-MM analysis was carried out (Supplementary Figure S6). Fifteen Hub genes were individually selected from the dark green and green modules, respectively (Supplementary Tables S5 and S6). An interaction network was constructed using the 30 Hub genes from both the dark green and green modules in relation to the total chlorophyll content, chlorophyll a content, and chlorophyll b content (with|Correlation| > 0.53 and *p*-value < 0.05). The findings revealed that the *BoOPR* gene has a positive regulatory effect on the total chlorophyll content, chlorophyll a content, and chlorophyll b content. Thus, this gene likely plays a significant role in chlorophyll metabolism (Figure 11A) (Supplementary Table S7). In the green module, twenty Hub genes were screened (Supplementary Table S6). An interaction network was created using these 20 Hub genes and five types of anthocyanin compounds (|Correlation| > 0.70, *p*-value < 0.05). The results indicated that two genes, *BoPAL7* and *BoLBD18*, regulate most of the anthocyanin compounds, with *BoPAL7* having a more extensive regulatory effect. Therefore, these two genes might contribute to anthocyanin synthesis (Figure 11B) (Supplementary Table S8).

### 2.10. Confirmation of Differentially Expressed Genes by Quantitative Real-Time PCR Analysis

**Figure 12:**
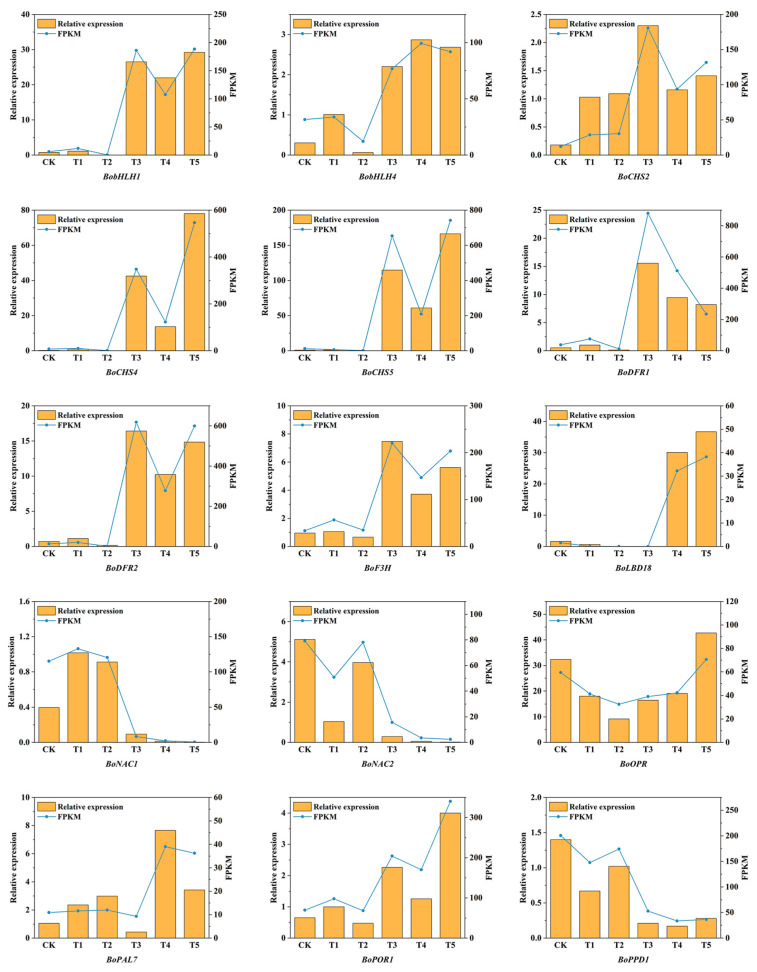
qRT-PCR validation of 15 DEGs in six broccoli varieties.

To validate the accuracy of the transcriptome sequencing results, this study randomly selected 15 genes with relatively high expression levels and related to the pigment content in florets for qRT-PCR verification. These genes included transcription factors and Hub genes associated with the pigment content in florets. The results of real-time fluorescence quantitative PCR were basically consistent with those of the transcriptome sequencing, demonstrating the high reliability of this sequencing (Figure 12).

## 3. Discussion

### 3.1. Analysis of Pigment Accumulation in Broccoli Florets with Different Colors

Studies on *Brassica* crops have indicated that chlorophyll, anthocyanin, and carotenoid are the major pigments determining the color diversity of *Brassica* plants [5]. Among them, the presence of chlorophyll is the main reason for the green color of plant tissues [43]. Anthocyanin can make plants appear red, purple, blue, and other colors, while carotenoid can cause plants to exhibit yellow, orange, pink, and other colors [44,45]. The floret color of ‘ZXQH62’ (CK) is normal green, that of ‘ZXQH67’ (T1) is yellow-green, that of ‘ZXQH27’ (T2) is light green, that of ‘ZXQH37’ (T3) is gray-green, that of ‘ZXQH71’ (T4) is blue-green, and that of ‘ZXQH42’ (T5) is dark blue-green. The color difference values and pigment contents of the florets at the harvest stage of the six materials were measured. The results showed that the chlorophyll content in the florets of CK, T1, and T2 was significantly higher than that of carotenoid, and there was no significant difference in the flavonoid content. In addition, the chlorophyll a content in T1 and T2 was significantly lower than that in CK, indicating that chlorophyll (chlorophyll a) was the main pigment component in the florets of CK, T1, and T2. Yu Hui et al. measured and analyzed the chlorophyll content in the florets of light green, green, gray-green, and blue-green broccoli and found that the change in floret color was directly related to the chlorophyll content, which was consistent with the conclusion obtained in this study [46]. In summary, the decrease in chlorophyll a content might be the reason for the florets to be yellow-green and light green, and it was independent of the carotenoid content. The chlorophyll content and anthocyanin content in the florets of T4 and T5 were both significantly higher than those in CK. In addition, the chlorophyll content and anthocyanin content in the florets of T5 were significantly higher than those in T4. It was speculated that the blue-green florets might be mainly determined by chlorophyll a and anthocyanin, and the levels of these two pigments would affect the shade of the blue-green florets. This was similar to the research results of Takayuki Mizuno et al. [47]. Wax is an important structure covering the surface of broccoli florets, usually in the form of a gray-green or gray-white frost-like substance, and its content can affect the color of the florets [48,49]. In this study, the chlorophyll content, carotenoid content, and anthocyanin content of T3 were not significantly different from those of CK. Therefore, it was speculated that this layer of wax had a certain covering effect on the main pigment layer, which might be the reason for the gray-green color of the T3 florets. Moreover, based on GO and KEGG enrichment, the DEGs in the CK vs. T3 combination were enriched in pathways related to wax synthesis, such as the cell response to fatty acids, linolenic acid metabolism, and fatty acid biosynthesis, further indicating that the gray-green color of broccoli might be caused by wax.

### 3.2. Metabolic Differences of Anthocyanins in Different Broccoli Varieties

Anthocyanins represent a vital category within plant flavonoid compounds. Based on their structural differences, they can be classified into six major types: pelargonidin, cyanidin, peonidin, delphinidin, petunidin, and malvidin [50]. Different types of anthocyanins display slightly varying colors. Pelargonidin and its derivatives appear red or orange, cyanidin and its derivatives are predominantly brick-red or mauve, peonidin and its derivatives are red or blue-purple, and delphinidin and its derivatives are blue or purple [51,52]. In this study, five types of anthocyanins were detected in broccoli, namely cyanidin, delphinidin, pelargonidin, peonidin, and petunidin. Among them, cyanidin and delphinidin accounted for 75.86%, suggesting that they are likely the main anthocyanin types in broccoli. In most *Brassica* plants such as kale, Chinese cabbage, and cauliflower, the coloring of their leaves and other organs is also mainly achieved through the accumulation of cyanidin and delphinidin [53,54,55]. However, due to the lack of relevant research, which anthocyanin compounds contribute to the coloration of blue-green broccoli remains to be determined.

Peonidin-3-O-glucoside was not detected in the green (CK), yellow-green (T1), and light green (T2) florets but was present in relatively high amounts in the blue-green (T4 and T5) broccoli. This indicates that peonidin-3-O-glucoside is a primary source of the blue-green color in florets. The contents of most cyanidin, delphinidin-3-O-glucoside-5-O-galactoside, pelargonidin, and peonidin-3,5-O-diglucoside in T5 were significantly higher than those in T4. Also, peonidin and delphinidin, along with their derivatives, are blue-colored, suggesting that the amounts of delphinidin-3-O-glucoside-5-O-galactoside and peonidin-3,5-O-diglucoside can influence the shade of the blue-green florets. However, delphinidins (delphinidin-3-O glucoside and delphinidin-3-O-galactoside) are the predominant anthocyanins in the purple broccoli variety PB767 [23]. Cyanidin-3-O-glucoside and delphinidin-3-O-glucoside are the predominant anthocyanins in purple kale [56]. Moreover, pelargonidin-3-O-(6″-O-malonyl) galactoside was only detected in T5, indicating that this anthocyanin compound plays a crucial role in deepening the color of blue-green florets. These findings reveal, for the first time, the presence of peonidin in blue-green broccoli and suggest that the color shade may be related to delphinidin and peonidin, which is rather unusual among blue-green *Brassica* vegetables.

### 3.3. Analysis of Key Structural Genes in Chlorophyll Metabolism and Anthocyanin Synthesis in Broccoli Florets

The CHLG accomplishes the final step of chlorophyll biosynthesis by esterifying chlorophyllide, and the downregulation of this gene can impede the biosynthesis process of chlorophyll a, resulting in yellow leaves [57]. In this study, the *BoCHLG* gene was upregulated in CK but downregulated in T1 and T2, and the CHLG gene was positively correlated with the chlorophyll a content in CK, T1, and T2. Therefore, the downregulation of the CHLG gene blocked the synthesis of chlorophyll a, leading to a decrease in the chlorophyll a content in T1 and T2 and causing the florets to appear light green and yellow-green. Zhang et al. conducted a transcriptome analysis on chlorophyll-deficient mutant Chinese cabbages and normal Chinese cabbages, and the results showed that the expression of the CHLG gene was significantly downregulated in the mutant Chinese cabbages [58]. This was consistent with the conclusion regarding the cause of the low chlorophyll content in T1 and T2 in this study. The SGR catalyzes the formation of pheophorbide a from chlorophyllide, which is a crucial step in the chlorophyll degradation pathway. PPD is also a key enzyme in chlorophyll degradation metabolism, and its expression intensifies during leaf senescence, accelerating leaf senescence [59]. In T4 and T5, the expression level of *BoHEMF* was significantly higher than that in CK, while the expression levels of *BoSGR1, BoSGR2*, *BoPPD1*, and *BoNYC* were significantly lower than that in CK. It was speculated that the high chlorophyll content in T4 and T5 was not only due to the upregulation of synthesis genes but also possibly caused by the downregulation of chlorophyll degradation genes. This was consistent with the research results of Wang et al. in Chinese cabbages [9]. PAL is the initial enzyme for anthocyanin synthesis and is encoded by a multigene family [60]. In T4 and T5, the expression levels of the *BoPAL2*, *BoFLS3*, and *BoUGT1* genes were significantly higher than those in CK, indicating that the upregulation of these three genes might lead to an increase in the anthocyanin content in T4 and T5. This was similar to the results found by Gu et al. in the study of purple-leaved plum leaves, where the enhanced PAL activity was accompanied by an increase in anthocyanin content, and the leaves gradually turned purplish-red [61].

Most upstream genes in chlorophyll synthesis, such as *BoCHLI2, BoCHLD*, *BoCHLM*, *BoDVR*, and *BoCLH*, had significantly higher expression levels in T5 than in T4. Zhou et al. conducted transcriptome and metabolome analyses on eggplant peels of different colors, and the results showed that chlorophyll synthesis genes such as HEMA, CHLH, and CHLM had higher expression levels in green eggplants [62]. Therefore, the high expression of most upstream genes in chlorophyll synthesis in T5 might be the reason for its higher chlorophyll content than T4. Anthocyanins are produced from a branch of the phenylpropanoid and flavonoid pathways, and differences in the expression patterns of genes involved in phenylpropanoid or flavonoid biosynthesis can lead to the production of different types of anthocyanins [63]. In the initial step of the flavonoid pathway, the enzymes encoded by PAL, C4H, and 4CL sequentially catalyze the metabolism of phenylalanine to coumaroyl-CoA, which provides precursor compounds for flavonoid synthesis [64]. In this study, compared to T4, the *BoPAL4*, C4H, *Bo4CL3*, *BoCHS4*, *BoF3′H2*, and *BoFLS1* genes had higher expression levels in T5, and the high expression of genes such as PAL, C4H, and 4CL provided sufficient precursor compounds for flavonoid biosynthesis. Therefore, the upstream genes in anthocyanin synthesis played an important role in the accumulation of anthocyanins in the dark blue-green florets (T5).

### 3.4. Analysis of Transcription Factors Related to Chlorophyll Metabolism and Anthocyanin Synthesis in Broccoli Florets

WRKY family transcription factors can directly or indirectly regulate the chlorophyll content in leaves [65]. In this study, the expression levels of *BoWRKY2* and *BoWRKY5* in CK were significantly higher than those in T1, T2, T4, and T5, and they interacted with *BoCHLH*. However, it is worth noting that the expression levels of the transcription factors and the CHLH gene were inconsistent in CK compared to T1, T2, T4, and T5. It is speculated that WRKY family transcription factors may directly regulate the chlorophyll content in florets. It has been found that transcription factor families such as NAC and TCP can affect the color of *Brassica* plants by regulating key genes in chlorophyll metabolism [5]. In this study, the expression levels of all NAC homologous DEGs, *BoTCP2*, and *BoTCP3* in CK were significantly higher than those in T4 and T5, and they all interacted with *BoSGR1, BoSGR2*, and *BoNYC*. In addition, the expression levels of the transcription factors and the chlorophyll degradation genes were consistent in CK, T4, and T5. Therefore, NAC and TCP family transcription factors directly regulate the chlorophyll degradation genes SGR and NYC, resulting in a lower chlorophyll content in CK than in T4 and T5. Si et al. found that the NAC family transcription factor *BrJUB1* directly regulates the transcription of chlorophyll degradation genes such as *BrNYC1* and *BrSGR1* in Chinese cabbage [66]. Xu et al. found that *Brassica rapa BrTCP7* recognizes the promoter sequence of *BrRCCR* and upregulates its expression to accelerate chlorophyll degradation [67]. These are all consistent with the results of this study. In addition, the expression levels of *BoTCP1* and *BoTCP4* in T5 were significantly higher than those in T4, which may be genes that directly regulate the chlorophyll accumulation in T4 and T5.

During the anthocyanin biosynthesis process, most MYB transcription factors have a positive regulatory role and usually contain two motifs such as R2 and R3 or a single motif R3 [68]. Dubos et al. found in Arabidopsis that AtMYB11 and AtMYB12 could both regulate the structural genes CHS, CHI, F3H, etc. involved in anthocyanin synthesis, thus increasing the anthocyanin content in Arabidopsis [69]. This is consistent with the results of this study. That is, the expression levels of *BoMYB1* and *BoMYB2* in T5 were significantly higher than those in T4, and they both interacted with the anthocyanin synthesis genes *Bo4CL3* and *BoCHS4*, indicating that MYB family transcription factors directly regulate the anthocyanin synthesis genes 4CL and CHS, thus affecting the anthocyanin content in T4 and T5. bHLH transcription factors can participate in regulating multiple physiological pathways, and regulating anthocyanin synthesis is one of its most important functions [68]. The expression levels of *BobHLH2* and *BobHLH3* in T4 and T5 were significantly higher than those in CK, which may be genes that directly regulate the anthocyanin accumulation in T4 and T5. This is similar to the research results of Mushtaq et al. [70].

### 3.5. Analysis of Other Key Candidate Genes Related to Chlorophyll Metabolism and Anthocyanin Synthesis in Brassica napus

The formation of anthocyanins depends on glycosylation, hydroxylation, acylation, and methoxylation to maintain stability, and this process is controlled by some transporters and other proteins [71]. In this study, WGCNA was employed to screen for key genes related to chlorophyll and anthocyanin synthesis in broccoli florets, and genes with high correlations were selected to construct an interaction network diagram. The results showed that the *BoOPR* gene positively regulates the total chlorophyll content, chlorophyll a content, and chlorophyll b content. The annotation of the *BoOPR* gene is 12-oxophytodienoate reductase 3 (Supplementary Table S9). OPR3 is an enzyme mainly involved in the biosynthetic pathway of jasmonates (JAs). It reduces the stability of JAs through phosphorylation, thereby limiting JA biosynthesis [72]. Fang et al. found that jasmonic acid can promote the yellowing of broccoli by enhancing the expression of genes related to chlorophyll degradation [73]. This indicates that the *BoOPR* gene may be indirectly involved in chlorophyll metabolism in broccoli florets. The *BoPAL7* and *BoLBD18* genes regulate most anthocyanin compounds. The annotation of the *BoPAL7* gene is PAL (Supplementary Table S9). PAL is one of the initial enzymes in the anthocyanin synthesis pathway. Its function is to catalyze the formation of cinnamic acid from phenylalanine, and this process is an important step in anthocyanin biosynthesis [74]. Jun et al. demonstrated that there is a highly significant positive linear correlation between the anthocyanin content in purple cabbage and the expression level of the PAL gene, indicating that PAL is a key enzyme in the anthocyanin synthesis of purple cabbage [75]. This is consistent with the research results of Chen et al. [76]. Therefore, the *BoPAL7* gene plays a significant role in the anthocyanin biosynthesis process of broccoli. The annotation of the *BoLBD18* gene is LOB domain-containing protein 18 (Supplementary Table S9). Ahn et al. found that, in green- and purple-headed cabbages, the negative regulatory function of the anthocyanin synthesis negative regulatory transcription factor LBD37 was absent, thus affecting the expression of downstream genes *BoMYB114L* and *BoTT8*, resulting in a change in the color of the cabbage [77]. This is similar to the results of this study, suggesting that the *BoLBD18* gene may indirectly regulate anthocyanin biosynthesis in broccoli.

## 4. Materials and Methods

### 4.1. Plant Materials

Six broccoli varieties with different floret colors were used as experimental materials. Among them, CK (‘ZXQH62’) was of normal green color, T1 (‘ZXQH67’) was yellow-green, T2 (‘ZXQH27’) was light green, T3 (‘ZXQH37′) was gray-green, T4 (‘ZXQH71’) was blue-green, and T5 (‘ZXQH42’) was dark blue-green. The seeds of all these varieties were provided by Xiamen Zhongxia Vegetable Seeds Co., Ltd. (Xiamen, China). Detailed information regarding the varieties is provided in Supplementary Table S10. All samples were cultivated in a glass greenhouse at the Fujian Academy of Agricultural Sciences (Fuzhou, China) (latitude 26°7′50″ N, longitude 119°20′4″ E) under natural photoperiod conditions, with day/night temperatures of 25/15 °C and a relative humidity of 70%, from 23 August 2023 to 20 January 2024 using a completely randomized block design. An automatic irrigation system was installed in the experimental area, which could supply water and fertilizer according to the water requirements of the plants to ensure their normal growth. The field cultivation and management were the same as those in large-scale field production.

At the harvest stage (the standard for the harvest stage was that the florets were firm and the edges did not disperse), the flower buds of the six broccoli varieties were collected. Four plants were randomly selected from each of the six varieties, with three biological replicates for each variety. Before collecting the flower buds, the color difference values of the florets of each variety were measured using a colorimeter. The flower bud samples of each variety were divided into three sample groups and stored in an ultra-low temperature freezer at −80 °C for subsequent analysis. One sample group was used for the measurement of chlorophyll content and anthocyanin content, and the other two sample groups were used for metabolite analysis and transcriptome sequencing (qRT-PCR), respectively.

### 4.2. Determination of Color Difference Values of Florets

The color difference values of the florets of the six broccoli varieties at the harvest stage were measured using a colorimeter (HIGH-QUALITYCOLORIMETER NH 310) by Shenzhen ThreeNH Technology Co., Ltd. (Shenzhen, China). Four measurement points spaced 90° apart on the equatorial plane were selected, and the average value was taken as the color difference measurement value for the floret of a single plant. Three plants were selected for each variety, and the measurement was repeated three times. Among them, L* (lightness) represents black and white, a* (redness and greenness) represents red and green, and b* (yellowness and blueness) represents yellow and blue.

### 4.3. Determination of the Chlorophyll Content and Flavonoid Content in Florets

The chlorophyll content and carotenoid content were determined using the immersion extraction method [78].

The flavonoid content and anthocyanin content were determined using the improved mixed solution extraction method [79]. Weigh 0.1 g of fresh flower bud samples into a 5 mL centrifuge tube, add 2 mL of acidified methanol (1% HCl), and extract in the dark at 4 °C for 24 h. Then, take the supernatant and transfer it to a microplate reader to measure the absorbance at wavelengths of 530 and 325 nm.

### 4.4. Identification and Quantification of Anthocyanins

The sample preparation, identification, and quantification of anthocyanins were entrusted to Wuhan MetWare Biotechnology Co., Ltd. (Wuhan, China). (http://www.metware.cn, accessed on 16 April 2024) to be carried out according to standard procedures. The sample extracts were analyzed using a liquid chromatography-tandem mass spectrometry (LC-MS/MS) system. The standardized metabolite data of the six varieties were used to compare metabolites. Hierarchical cluster analysis (HCA) and principal component analysis (PCA) of the six samples were performed using the Complex Heatmap package in R software to study the metabolite differences among the six samples. Metabolites with significant regulation between groups were determined using the criteria of VIP ≥ 1, fold change ≥ 2, or fold change ≤ 0.5. The identified metabolites were annotated using the KEGG (Kyoto Encyclopedia of Genes and Genomes) database (http://www.metware.cn, accessed on 16 April 2024) and then mapped to the KEGG pathway database (http://www.kegg.jp/kegg/pathway.html, accessed on 16 April 2024).

### 4.5. RNA-Seq Analysis

Six samples of florets from broccoli varieties with different floret colors, collected in three biological replicates, were used for transcriptomic analysis. The total RNA in the broccoli florets was extracted using the RNA prep Pure Polysaccharide and Polyphenol Plant Total RNA Extraction Kit. After successful extraction of the total RNA, the concentration and integrity of the RNA were detected using a Qubit 4.0 fluorometer, a MD microplate reader, and a Qsep400 bioanalyzer. The RNA libraries that passed the quality inspection were sequenced on the Illumina platform. The original sequencing data were first processed by Trimmamotic 0.39 software to remove adapters and low-quality sequences. Subsequently, the obtained clean reads were aligned to the Boleracea reference genome (http://39.100.233.196:82/download_genome/Brassica_Genome_data/Braol_HDEM_V1.0/, accessed on 16 April 2024) using Hisat2, and the FPKM values of each gene were calculated using feature counts.

### 4.6. RNA-seq Data Analysis and Annotation

Differential expression analysis between sample groups was performed using DESeq2 software. Genes that met the thresholds of FDR < 0.05 and |log2Fold Change| ≥ 1 were defined as differentially expressed genes (DEGs), and GO functional annotation and KEGG enrichment analysis were carried out on the DEGs. Based on the GO and KEGG enrichment results and combined with the pathways related to pigment synthesis and metabolism in the KEGG, the DEGs involved in the pathways related to floret color formation were analyzed.

### 4.7. Weighted Gene Co-Expression Network Analysis

The weighted gene co-expression network analysis (WGCNA)was performed on all differentially expressed genes using the WGCNA R software package. The Pearson correlation between all genes was calculated to generate an adjacency matrix. Correlation analysis between identified modules and sample traits was carried out using all the genes in each module. Using Cytoscape 3.7.2 software (http://www.cytoscape.org, accessed on 16 April 2024), co-expression networks were constructed for Hub genes related to the chlorophyll content in significantly positive modules and for Hub genes related to the chlorophyll content, anthocyanin content, and anthocyanin metabolites.

### 4.8. Transcription Factor Analysis

To identify the transcription factors involved in chlorophyll and anthocyanin synthesis among the DEGs, the online website STRING (http://cn.string-db.org, accessed on 16 April 2024) was used to analyze the protein–protein interaction network between the DEGs related to chlorophyll and anthocyanin pathways and putative transcription factors. During the analysis, the protein sequences encoded by the putative transcription factors and the DEGs related to the chlorophyll and anthocyanin pathways were uploaded under the ‘Multiple sequences’ option, respectively, and the species was set as *Brassica oleracea var. italica*, with the other options set to default. The best matching result for each sequence was selected for drawing the protein interaction network. Finally, the data analyzed by STRING were imported into Cytoscape 3.7.2 software (http://www.cytoscape.org, accessed on 16 April 2024) for beautification.

### 4.9. qRT-PCR Analysis

The expression levels of 15 DEGs in the six varieties were analyzed using real-time fluorescence quantitative PCR (qRT-PCR) technology. The first-strand cDNA and qRT-PCR reactions were synthesized using kits from Nanjing Vazyme Biotech Co., Ltd. (Nanjing, China) (https://bio.vazyme.com/, accessed on 16 April 2024). The primers for qRT-PCR were designed by Shangya Biotech Co. (http://www.zjsyswjs.com/, accessed on 16 April 2024). The sequence-specific primers used for qRT-PCR, including those for the *Actin* gene and the 15 selected genes, are listed in Supplementary Table S11. Each sample was analyzed with three technical replicates, and the relative expression levels were normalized to the *Actin* gene and calculated using the 2^−ΔΔCt^ method [80].

### 4.10. Data Analysis

Microsoft Excel 2019 was used for data analysis and creating data charts. SPSS 26.0 was used for statistical analysis, correlation analysis, and significance test of differences (*p* < 0.05). Origin 2018 was used for graph drawing.

## 5. Conclusions

This study, through integrated transcriptomic and targeted metabolomic analyses, preliminarily revealed the molecular mechanisms underlying floret color formation in broccoli, demonstrating that the diversity of floret color is determined by the differential expression of key genes and the accumulation levels of metabolites in chlorophyll and anthocyanin metabolic pathways. Notably, the significant accumulation of peonidin-3-O-glucoside, delphinidin-3-O-glucoside, and peonidin-3,5-O-diglucoside in blue-green florets, along with the high expression of the chlorophyll synthesis gene *BoCHLG* in green florets, provides new insights into the molecular basis of floret color formation. The findings hold significant implications for molecular breeding, as the screening and regulation of key genes related to pigment metabolism (e.g., *BoCHLG*, *BoSGR*, and *BoPAL*) can enable the targeted improvement of broccoli color traits, leading to the development of new varieties with greater market appeal. Additionally, the candidate genes identified in this study offer valuable genetic resources for marker-assisted selection (MAS), accelerating the breeding process. Furthermore, the results provide potential raw materials for the development of functional foods and natural pigment extraction, highlighting broad application prospects. Floret color not only influences the commercial value of broccoli but may also significantly impact its postharvest quality. Studies suggest that anthocyanins, with their strong antioxidant activity, may extend the shelf life and enhance the nutritional value of broccoli. Moreover, darker florets (e.g., blue-green) may be more appealing to consumers, thereby increasing market competitiveness. Future research should further validate the functions of key genes; explore the regulatory mechanisms of environmental factors; investigate the relationship between floret color and postharvest quality (e.g., antioxidant properties, shelf life, and consumer preference); and apply the findings to practical breeding efforts to develop broccoli varieties with superior color traits and nutritional value. In summary, this study provides novel insights into the molecular mechanisms of floret color formation in broccoli and lays an important foundation for molecular breeding and postharvest quality improvement. Future research will further advance scientific progress and practical applications in this field.

## Figures and Tables

**Table 1:** Changes in the chlorophyll and carotenoid contents of the flower heads of six broccoli varieties.

Varieties	Chlorophyll a mg g^−1^ FW	Chlorophyll b mg g^−1^ FW	Total Chlorophyll mg g^−1^ FW	Carotenoid mg g^−1^ FW	Chlorophyll a/b	Chlorophyll/Carotenoid
CK	0.15 ± 0.01 c	0.07 ± 0.01 c	0.22 ± 0.02 c	0.03 ± 0.00 c	2.36 ± 0.06 a	0.13 ± 0.01 bc
T1	0.05 ± 0.01 e	0.02 ± 0.01 d	0.07 ± 0.01 d	0.01 ± 0.00 e	2.43 ± 0.67 a	0.17 ± 0.05 a
T2	0.13 ± 0.01 d	0.06 ± 0.01 c	0.19 ± 0.01 c	0.02 ± 0.00 d	2.10 ± 0.08 a	0.11 ± 0.01 c
T3	0.15 ± 0.01 cd	0.06 ± 0.01 c	0.20 ± 0.01 c	0.03 ± 0.00 c	2.54 ± 0.14 a	0.14 ± 0.00 abc
T4	0.25 ± 0.02 b	0.10 ± 0.02 b	0.36 ± 0.03 b	0.06 ± 0.00 b	2.47 ± 0.24 a	0.16 ± 0.02 ab
T5	0.43 ± 0.00 a	0.19 ± 0.01 a	0.62 ± 0.02 a	0.10 ± 0.00 a	2.26 ± 0.11 a	0.16 ± 0.00 ab

The average value of three replicates ± SE. In the same column, the same letters indicate no significant difference, while different letters indicate a significant difference (*p* < 0.05). The same applies hereinafter.

**Table 2:** Transcription factors related to the synthesis pathways of chlorophyll and anthocyanin.

TF Family	Gene Symbol
WRKY	*BoWRKY1*
	*BoWRKY2*
	*BoWRKY3*
	*BoWRKY4*
	*BoWRKY5*
NAC	*BoNAC1*
	*BoNAC2*
	*BoNAC3*
TCP	*BoTCP1*
	*BoTCP2*
	*BoTCP3*
	*BoTCP4*
bHLH	*BobHLH1*
	*BobHLH2*
	*BobHLH3*
	*BobHLH4*
MYB	*BoMYB1*
	*BoMYB2*

## Data Availability

Data are contained within the article and Supplementary Materials.

## Supplementary Materials

The following supporting information can be downloaded at https://www.mdpi.com/article/10.3390/plants14060849/s1, Table S1. Statistical analysis of the filtration and alignment of the transcriptome sequencing data; Table S2. Differentially expressed gene IDs and gene symbols related to the chlorophyll metabolism pathway; Table S3. Differentially expressed gene IDs and gene symbols related to the anthocyanin metabolism pathway; Table S4. Gene IDs and gene symbols of transcription factors associated with the chlorophyll and anthocyanin biosynthesis pathways; Table S5. The kWithin values of the 15 screened Hub genes in the darkgreen module; Table S6. The kWithin values of the 20 screened Hub genes in the green module; Table S7. The interactions between the screened Hub genes and the contents of total chlorophyll, chlorophyll a, and chlorophyll b; Table S8. Interactions between the screened Hub genes and five anthocyanin—type compounds; Table S9. Functional annotation of the screened Hub genes; Table S10. Detailed Information of the Variety; Table S11. The primer sequences of the differentially expressed genes; Figure S1. Principal component analysis plot of the samples from 18 broccoli flower buds; Figure S2. GO classification diagrams of differentially expressed genes. (A) GO classification diagram of DEGs in the CK vs T1 vs T2 combination; (B) GO classification diagram of DEGs in the CK vs T3 combination; (C) GO classification diagram of DEGs in the CK vs. T4 vs. T5 combination; (D) GO classification diagram of DEGs in the T4 vs. T5 combination; Figure S3. GO enrichment bar charts of differentially expressed genes. (A) GO enrichment bar chart of DEGs in the CK vs. T1 vs. T2 combination; (B) GO enrichment bar chart of DEGs in the CK vs. T3 combination; (C) GO enrichment bar chart of DEGs in the CK vs. T4 vs. T5 combination; (D) GO enrichment bar chart of DEGs in the T4 vs. T5 combination; Figure S4. Scatter plots of KEGG enrichment for DEGs. (A) Scatter plot of KEGG enrichment for DEGs in the CK vs T1 vs T2 combination; (B) Scatter plot of KEGG enrichment for DEGs in the CK vs T3 combination; (C) Scatter plot of KEGG enrichment for DEGs in the CK vs T4 vs T5 combination; (D) Scatter plot of KEGG enrichment for DEGs in the T4 vs T5 combination; Figure S5. Weighted gene co-expression network analysis of 10,876 DEGs). (A) KEGG enrichment bubble plot of genes in the grey-green module; (B) KEGG enrichment bubble plot of genes in the green module; (C) Heatmap of co-expressed genes in the darkgreen module; (D) Heatmap of co-expressed genes in the green module; Figure S6. Screening of Hub genes. (A) GS-MM analysis of the grey-green module in total chlorophyll content; (B) GS-MM analysis of the green module in total chlorophyll content; (C) GS-MM analysis of the grey-green module in chlorophyll a content; (D) GS-MM analysis of the green module in chlorophyll a content; (E) GS-MM analysis of the green module in anthocyanin content.
