# Supplementary material for: Preliminary Analysis of the Formation Mechanism of Floret Color in Broccoli (Brassica oleracea L var. italica) Based on Transcriptomics and Targeted Metabolomics
Source: Plants (Basel). 2025 Mar 8;14(6):849. doi: 10.3390/plants14060849 (PMC11945052; doi:10.3390/plants14060849)
Supplement: Supplementary file 1 [file plants-14-00849-s001.zip › plants-3502658-supplementary.pdf]

Supplementary Table S1. Statistical analysis of the filtration and alignment of the transcriptome sequencing data.

Sample	Raw Reads	Clean Reads	Reads mapped	Q20(%)	Q30(%)	GC Content(%)
CK-1	71365858	67975372	63018873(92.71%)	98.53	95.82	46.83
CK-2	64499506	61066916	56523867(92.56%)	98.38	95.38	46.84
CK-3	70576740	66494936	62075579(93.35%)	98.70	95.83	46.97
T1-1	61437400	58777180	54530896(92.78%)	98.85	96.25	46.79
T1-2	66924950	62913134	57777981(91.84%)	98.29	95.28	46.89
T1-3	62548856	58360742	53445853(91.58%)	98.30	95.31	46.91
T2-1	60439608	56612366	51653277(91.24%)	98.37	95.49	46.88
T2-2	44455312	41304966	37595993(91.02%)	98.20	95.02	46.98
T2-3	62037584	58186206	53014725(91.11%)	98.24	95.14	46.89
T3-1	64194380	60565414	56348130(93.04%)	98.50	95.74	47.02
T3-2	69097942	65424396	60800285(92.93%)	98.51	95.79	47.02
T3-3	61359516	57829630	53733341(92.92%)	98.45	95.59	47.05
T4-1	91360520	85816162	80213629(93.47%)	98.73	95.95	47.03
T4-2	105045520	97919182	90774395(92.70%)	98.28	95.25	47.08
T4-3	82376568	75103994	69562398(92.62%)	98.48	95.77	47.09
T5-1	75120934	69721052	64195159(92.07%)	98.19	95.00	47.45
T5-2	101750382	92723146	85674286(92.40%)	98.41	95.54	47.61

Supplementary Table S2. Differentially expressed gene IDs and gene symbols related to the chlorophyll metabolism pathway.

Gene ID	Gene symbol
BolC8t47883H	*BoHEMA*
BolC3t18541H	*BoHEML1*
BolC9t53854H	*BoHEML2*
BolC6t39425H	*BoHEMB*
BolC2t06308H	*BoHEMC1*
BolC9t59612H	*BoHEMC2*
BolC4t28337H	*BoHEME*
BolC5t28975H	*BoHEMF*
BolC9t59131H	*BoHEMY*

BolC1t01217H	*BoCHLI1*
BolC2t10724H	*BoCHLI2*
BolC3t13211H	*BoCHLH*
BolC5t29422H	*BoCHLD*
BolC7t45474H	*BoCHLI3*
BolC1t01979H	*BoCHLM*
BolC6t37555H	*BoACSF*
BolC1t02306H	*BoPOR1*
BolC7t46028H	*BoPOR2*
BolC9t58650H	*BoDVR*
BolC5t30519H	*BoCLH*
BolC1t02697H	*BoCHLG*
BolC3t15877H	*BoSGR1*
BolC7t45722H	*BoSGR2*
BolC6t35201H	*BoCAO*
BolC1t02539H	*BoPPD1*
BolC2t07458H	*BoPPD2*
BolC8t48387H	*BoPPD3*
BolC4t25835H	*BoNYC*

Supplementary Table S3. Differentially expressed gene IDs and gene symbols related to the anthocyanin metabolism pathway.

Gene ID	Gene symbol
BolC3t17676H	*BoPAL1*
BolC4t22683H	*BoPAL2*
BolC5t33481H	*BoPAL3*
BolC6t36657H	*BoPAL4*
BolC7t40642H	*BoPAL5*
BolC8t46904H	*BoPAL6*
BolC4t23544H	*BoC4H1*
BolC4t27445H	*BoC4H2*
BolC2t12319H	*Bo4CL1*
BolC6t38989H	*Bo4CL2*
BolC9t54487H	*Bo4CL3*
BolC2t06596H	*BoCHS1*
BolC3t13229H	*BoCHS2*
BolC3t15595H	*BoCHS3*
BolC3t20825H	*BoCHS4*
BolC4t28136H	*BoCHS5*
BolC7t46509H	*BoCHS6*
BolC9t59153H	*BoCHS7*
BolC6t37453H	*BoCHI1*
BolC9t59906H	*BoCHI2*

BolC8t50265H	*BoF3H*
BolC3t20722H	*BoF3'H1*
BolC9t59639H	*BoF3'H2*
BolC1t03277H	*BoFLS1*
BolC2t12354H	*BoFLS2*
BolC9t59578H	*BoFLS3*
BolC1t00338H	*BoDFR1*
BolC3t20308H	*BoDFR2*
BolC4t25146H	*BoANS1*
BolC8t50080H	*BoANS2*
BolC3t14148H	*BoUGT1*
BolC3t14149H	*BoUGT2*
BolC5t32652H	*BoUGT3*
BolC9t56424H	*BoUGT4*
BolC8t48098H	*BoUGT5*

Supplementary Table S4. Gene IDs and gene symbols of transcription factors associated with the chlorophyll and anthocyanin biosynthesis pathways.

Gene ID	Gene symbol
BolC6t40382H	*BoWRKY1*
BolC2t09701H	*BoWRKY2*
BolC6t37525H	*BoWRKY3*

BolC7t46336H	*BoWRKY4*
BolC4t28351H	*BoWRKY5*
BolC6t39391H	*BoNAC1*
BolC6t38805H	*BoNAC2*
BolC4t26184H	*BoNAC3*
BolC6t39422H	*BoTCP1*
BolC2t08144H	*BoTCP2*
BolC7t44125H	*BoTCP3*
BolC6t38779H	*BoTCP4*
BolC7t41091H	*BobHLH1*
BolC5t29220H	*BobHLH2*
BolC4t23452H	*BobHLH3*
BolC4t27562H	*BobHLH4*
BolC2t07665H	*BoMYB1*
BolC9t57583H	*BoMYB2*

Supplementary Table S5. The kWithin values of the 15 screened Hub genes in the darkgreen module.

Gene ID	Gene symbol	Module Colors	KWithin
BolC1t01503H	*Bo1g015030*	darkgreen	22.06
BolC1t02206H	*BoglpQ*	darkgreen	21.00
BolC2t07095H	*Bo2g070950*	darkgreen	24.17
BolC2t07458H	*BoPPD2*	darkgreen	23.70
BolC2t08203H	*BoLOX2S*	darkgreen	20.15
BolC2t08583H	*BoXYL1*	darkgreen	22.59
BolC2t08850H	*BoJAZ1*	darkgreen	23.31
BolC3t17161H	*BometK*	darkgreen	19.68
BolC3t17841H	*BoOPR*	darkgreen	21.81
BolC3t18276H	*BoSCYL1*	darkgreen	19.47

BolC6t39537H	*BoJAZ2*	darkgreen	24.51
BolC6t40051H	*Bo6g400510*	darkgreen	24.12
BolC7t46032H	*Bo7g460320*	darkgreen	23.30
novel.195	*Bo195*	darkgreen	22.38
novel.2480	*Bo2480*	darkgreen	22.15

Supplementary Table S6. The kWithin values of the 20 screened Hub genes in the green module.

Gene ID	Gene symbol	Module Colors	KWithin
BolC1t04840H	*Bo1g048400*	green	81.02
BolC1t04902H	*Bo1g049020*	green	77.78
BolC1t05347H	*BoZNHIT1*	green	77.42
BolC2t09007H	*Bo2g090070*	green	82.48
BolC2t09225H	*BoPAL7*	green	76.85
BolC2t10294H	*BoGBA2-1*	green	80.10
BolC2t10295H	*BoGBA2-2*	green	78.99
BolC2t10316H	*Bo2g103160*	green	80.45
BolC2t10366H	*BoU2AF1*	green	81.37
BolC2t10471H	*Bo2g104710*	green	75.05
BolC2t10681H	*BoMAK*	green	78.33
BolC3t16326H	*BoLBD18*	green	79.10
BolC5t29184H	*BoFRK1*	green	75.49
BolC5t33757H	*Bo5g337570*	green	80.98
BolC6t38213H	*BoGST*	green	77.87
BolC6t39949H	*Bo6g399490*	green	82.52
BolC8t47230H	*Bo8g472300*	green	75.99
BolC8t48246H	*Bo8g482460*	green	76.17
novel.3917	*BoPLRG1*	green	77.28
novel.965	*Bo965*	green	76.52

Supplementary Table S7. The interactions between the screened Hub genes and the contents of total chlorophyll, chlorophyll a, and chlorophyll b.

Omics 1	Omics 2	Correlation
BoOPR	Total chlorophyll	0.55
BoOPR	Chlorophyll a	0.55
BoOPR	Chlorophyll b	0.53

Supplementary Table S8. Interactions between the screened Hub genes and five anthocyanin - type compounds.

Omics 1	Omics 2	Omics 2 Compounds	Correlation
BoPAL7	Anthocyanidin_171	Cyanidin-3-O-(sinapoyl) xyloside-5-O-glucoside-7-O-glucoside	-0.71
BoPAL7	Anthocyanidin_10	Cyanidin-3-O-galactoside	-0.87
BoPAL7	Anthocyanidin_15	Cyanidin-3-O-sophoroside	-0.86
BoLBD18	Anthocyanidin_11	Cyanidin-3-O-glucoside	-0.71
BoLBD18	Anthocyanidin_172	Cyanidin-3-O-(feruloyl) glucoside-5-O-diglucoside	-0.72

Supplementary Table S9. Functional annotation of the screened Hub genes.

Gene	Annotation
BoOPR	12-oxophytodienoate reductase 3
BoPAL7	Phenylalanine ammonia-lyase
BoLBD18	LOB domain-containing protein 18

Supplementary Table S10. Detailed Information of the Variety.

Plot ID	Variety ID	Lineage Number	Floret Color	Leaf Color
ZXQH62	G93018	G93018-5	Green	Bluish-green
ZXQH67	G9906	G9906-5	Yellow-green	Bluish-green
ZXQH27	MS97125	MS97125-5	Light green	Bluish-green
ZXQH37	G03029	G03029-5	Grayish-green	Grayish-green
ZXQH71	GB13	GB13-5	Bluish-green	Bluish-green
ZXQH42	G1060	G1060-5	Dark Bluish -green	Bluish-green

Supplementary Table S11. The primer sequences of the differentially expressed genes.

Gene symbol	Primer Sequences（5′-3′）
BoDFR1	F：CGTTACCCATCACTCCATATCC<br>R：AATGCAGTCATCGAACATCTCTT
BoPOR1	F：TACCCTCTTCCCTCCGTTTC<br>R：CGCTTGCTTCTTCCGATAACT
BoPPD1	F：GGCTTTGAACGGTGCTGATA<br>R：ACTTCTCCACTACACGGTCTT
BoCHS2	F：CTACTTCCGCATCACCAACAG<br>R：ACCACCACGATGTCTTGTCT
BoDFR2	F：TCACTGTTCTACCATCCTTCCT<br>R：CCATCTGTCCATGCCATTGAA
BoCHS4	F：ATCTTGTTGGTGTTGCCTTGT<br>R：AGTTTATCCCTTGTTCCGTTAGC
BobHLH4	F：CCATCAAGGTTGTTCAGAAGAAGA<br>R：GCAATTCCACTCGCATACACA
BoCHS5	F：GAAGGGCGTTGGTGGATTATG<br>R：GAGGCTACGGAGGAGAAGAC
BoNAC2	F：AGTACCGTCTCCACGATTCA<br>R：CTTCCCTGCTCCTCTCTTCT
BoNAC1	F：TTCATAGTCCTGATCCGAACATTG

	R：ATACTAGCCTGACGATGATGGT
BobHLH1	F：GATTGTAGCGACCAGATTGATGA<br>R：CTTCTTCCTCCTCCGTCTCT
BoF3H	F：ACTTCTTCGCCTTACCTCCT<br>R：CTCTCCAATCTTGAACAGACTCTC
Actin	F：TGGACTACGAGCAGGAGATG<br>R：AACGATGGCTGGAACAAGAC
BoOPR	F：GCTCAACAAGTTACAGGACGATAC<br>R：TGCCTTGCCTTCCAGATTCT
BoPAL7	F：GACAGGTTCAGGTTCCATAGC<br>R：AGCAGTGTGGTCAGTGATAGT
BoLBD18	F：AGCCACAACACTTCAGATTCTC<br>R：TCCACTAGCACACTTCCTTCT

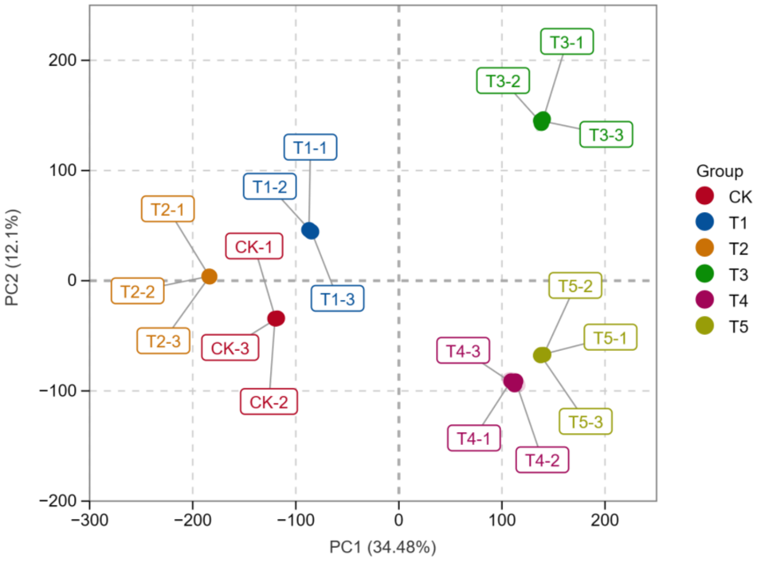

Supplementary Figure S1. Principal component analysis plot of the samples from 18 broccoli flower buds.

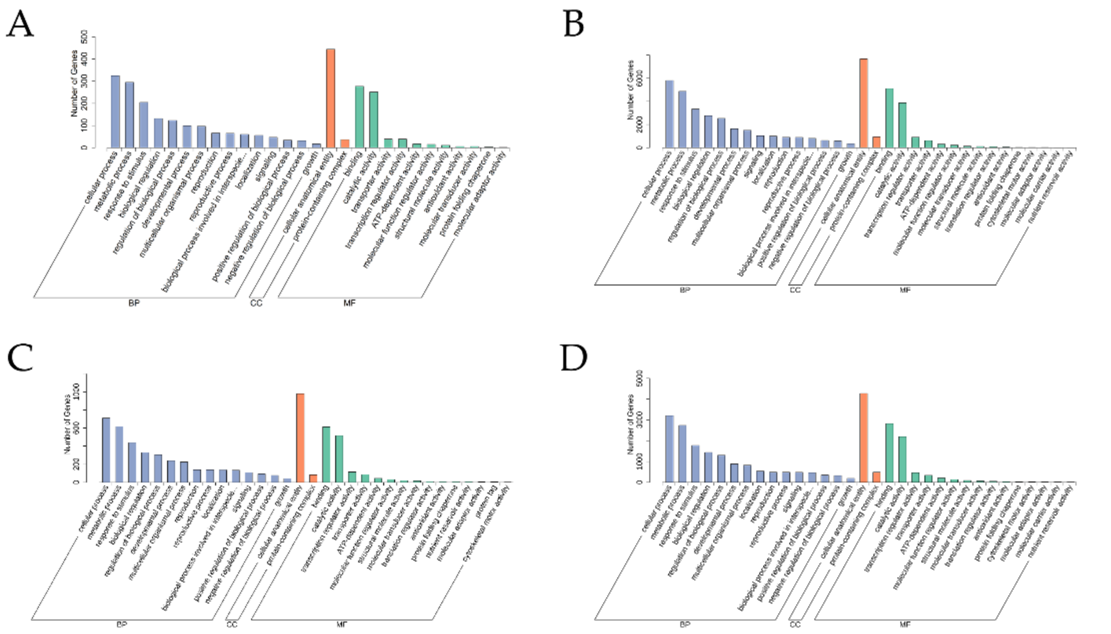

Supplementary Figure S2. GO classification diagrams of differentially expressed genes. (A) GO classification diagram of DEGs in the CK vs T1 vs T2 combination; (B) GO classification diagram of DEGs in the CK vs T3 combination; (C) GO classification diagram of DEGs in the CK vs T4 vs T5 combination; (D) GO classification diagram of DEGs in the T4 vs T5 combination.

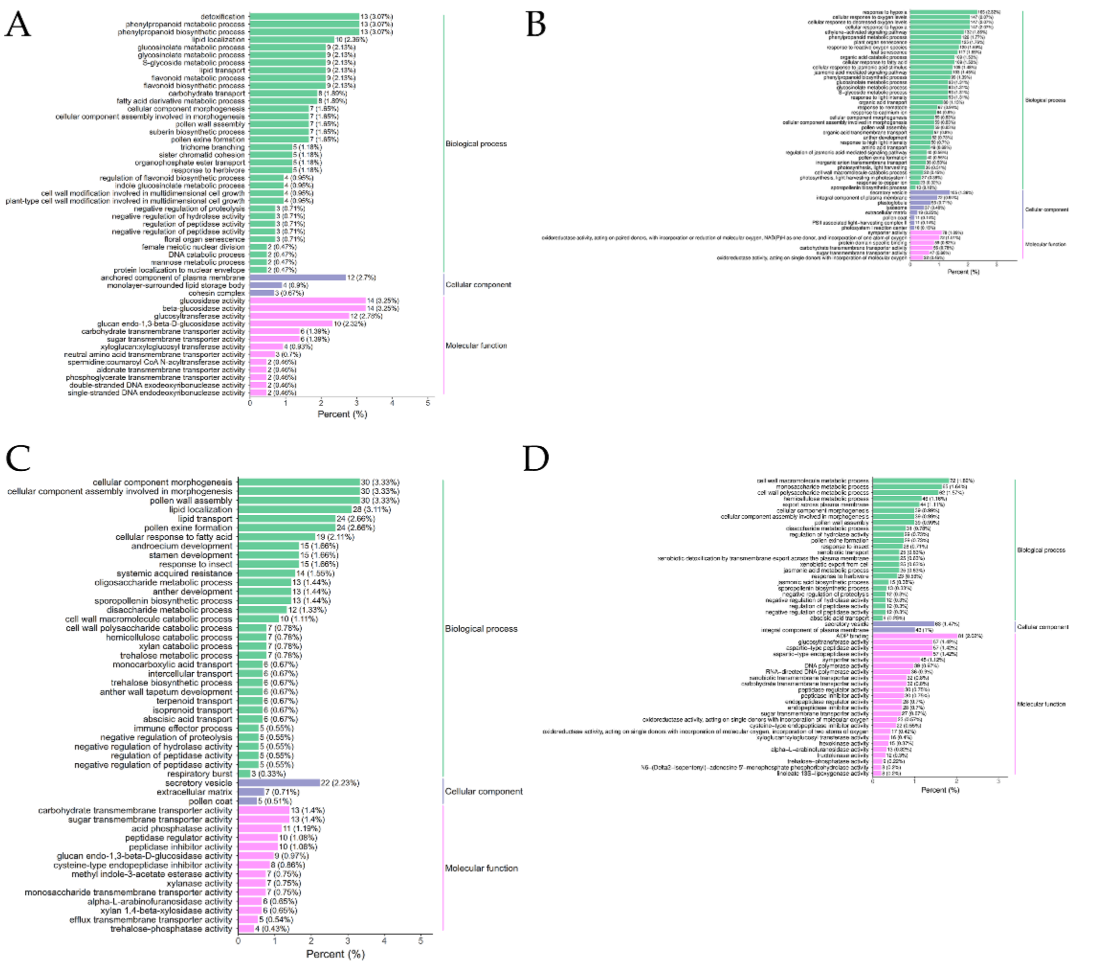

Supplementary Figure S3. GO enrichment bar charts of differentially expressed genes. (A) GO enrichment bar chart of DEGs in the CK vs T1 vs T2 combination; (B) GO enrichment bar chart of DEGs in the CK vs T3 combination; (C) GO enrichment bar chart of DEGs in the CK vs T4 vs T5 combination; (D) GO enrichment bar chart of DEGs in the T4 vs T5 combination.

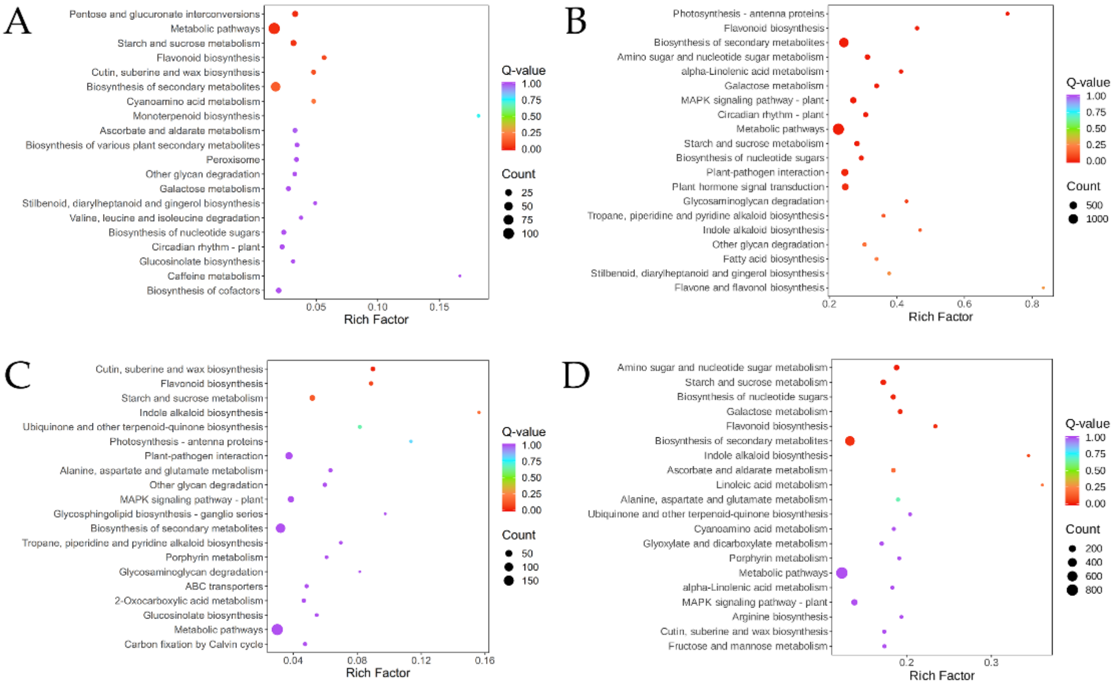

Supplementary Figure S4. Scatter plots of KEGG enrichment for DEGs. (A) Scatter plot of KEGG enrichment for DEGs in the CK vs T1 vs T2 combination; (B) Scatter plot of KEGG enrichment for DEGs in the CK vs T3 combination; (C) Scatter plot of KEGG enrichment for DEGs in the CK vs T4 vs T5 combination; (D) Scatter plot of KEGG enrichment for DEGs in the T4 vs T5 combination.

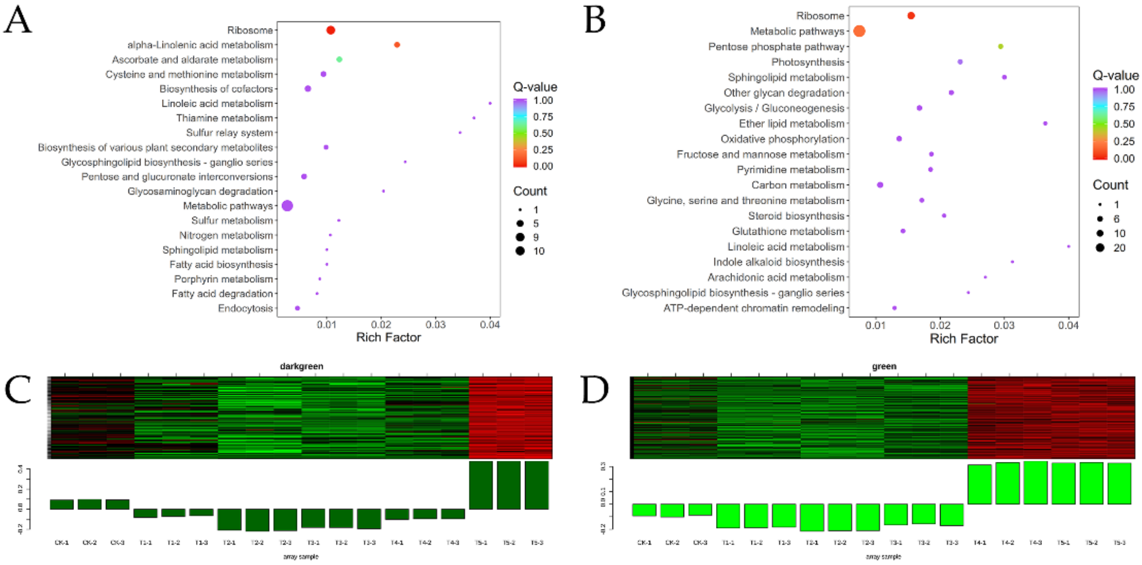

Supplementary Figure S5. Weighted gene co - expression network analysis of 10,876 DEGs). (A) KEGG enrichment bubble plot of genes in the grey - green module; (B) KEGG enrichment bubble plot of genes in the green module; (C) Heatmap of co - expressed genes in the darkgreen module; (D) Heatmap of co - expressed genes in the green module.

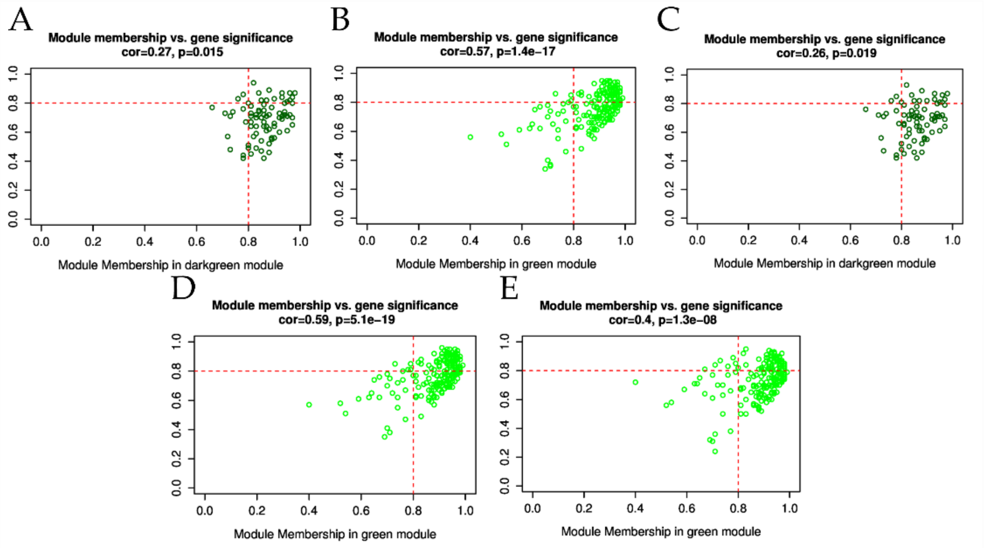

Supplementary Figure S6. Screening of Hub genes. (A) GS - MM analysis of the grey - green module in total chlorophyll content; (B) GS - MM analysis of the green module in total chlorophyll content; (C) GS - MM analysis of the grey - green module in chlorophyll a content; (D) GS - MM analysis of the green module in chlorophyll a content; (E) GS - MM analysis of the green module in anthocyanin content.
